# The Effect of Sitagliptin on Carotid Artery Atherosclerosis in Type 2 Diabetes: The PROLOGUE Randomized Controlled Trial

**DOI:** 10.1371/journal.pmed.1002051

**Published:** 2016-06-28

**Authors:** Jun-ichi Oyama, Toyoaki Murohara, Masafumi Kitakaze, Tomoko Ishizu, Yasunori Sato, Kazuo Kitagawa, Haruo Kamiya, Masayoshi Ajioka, Masaharu Ishihara, Kazuoki Dai, Mamoru Nanasato, Masataka Sata, Koji Maemura, Hirofumi Tomiyama, Yukihito Higashi, Kohei Kaku, Hirotsugu Yamada, Munehide Matsuhisa, Kentaro Yamashita, Yasuko K. Bando, Naoki Kashihara, Shinichiro Ueda, Teruo Inoue, Atsushi Tanaka, Koichi Node, PROLOGUE Study Investigators

**Affiliations:** 1 Department of Cardiovascular Medicine, Saga University, Saga, Japan; 2 Department of Cardiology, Nagoya University Graduate School of Medicine, Nagoya, Japan; 3 Department of Clinical Medicine and Development, National Cerebral and Cardiovascular Center, Osaka, Japan; 4 Department of Clinical Laboratory Medicine, Faculty of Medicine, University of Tsukuba, Tsukuba, Japan; 5 Department of Global Clinical Research, Graduate School of Medicine, Chiba University, Chiba, Japan; 6 Department of Neurology, Tokyo Women’s Medical University, Tokyo, Japan; 7 Division of Cardiology, Japanese Red Cross Nagoya Daiichi Hospital, Nagoya, Japan; 8 Department of Cardiovascular Internal Medicine, Tosei General Hospital, Seto, Japan; 9 Division of Cardiovascular Medicine and Coronary Heart Disease, Hyogo College of Medicine, Nishinomiya, Japan; 10 Department of Cardiology, Hiroshima City Hospital, Hiroshima, Japan; 11 Cardiovascular Center, Japanese Red Cross Nagoya Daini Hospital, Nagoya, Japan; 12 Department of Cardiovascular Medicine, Institute of Biomedical Sciences, Tokushima University Graduate School, Tokushima, Japan; 13 Department of Cardiovascular Medicine, Graduate School of Biomedical Sciences, Nagasaki University, Nagasaki, Japan; 14 Department of Cardiology, Tokyo Medical University, Tokyo, Japan; 15 Department of Cardiovascular Regeneration and Medicine, Research Institute for Radiation Biology and Medicine, Hiroshima University, Hiroshima, Japan; 16 Department of Internal Medicine, Kawasaki Medical School, Kurashiki, Japan; 17 Department of Cardiovascular Medicine, Tokushima University Hospital, Tokushima, Japan; 18 Diabetes Therapeutics and Research Center, Tokushima University, Tokushima, Japan; 19 Department of Cardiology, Nagoya University Graduate School of Medicine and National Hospital Organization Nagoya Medical Center, Nagoya, Japan; 20 Department of Nephrology and Hypertension, Kawasaki Medical School, Kurashiki, Japan; 21 Department of Clinical Pharmacology and Therapeutics, University of the Ryukyus, Nishihara, Japan; 22 Department of Cardiovascular Medicine, Dokkyo Medical University, Mibu, Japan; Stanford University, UNITED STATES

## Abstract

**Background:**

Experimental studies have suggested that dipeptidyl peptidase-4 (DPP-4) inhibitors provide cardiovascular protective effects. We performed a randomized study to evaluate the effects of sitagliptin added on to the conventional therapy compared with conventional therapy alone (diet, exercise, and/or drugs, except for incretin-related agents) on the intima-media thickness (IMT) of the carotid artery, a surrogate marker for the evaluation of atherosclerotic cardiovascular disease, in people with type 2 diabetes mellitus (T2DM).

**Methods and Findings:**

We used a multicenter PROBE (prospective, randomized, open label, blinded endpoint) design. Individuals aged ≥30 y with T2DM (6.2% ≤ HbA1c < 9.4%) were randomly allocated to receive either sitagliptin (25 to 100 mg/d) or conventional therapy. Carotid ultrasound was performed at participating medical centers, and all parameters were measured in a core laboratory. Of the 463 enrolled participants with T2DM, 442 were included in the primary analysis (sitagliptin group, 222; conventional therapy group, 220). Estimated mean (± standard error) common carotid artery IMT at 24 mo of follow-up in the sitagliptin and conventional therapy groups was 0.827 ± 0.007 mm and 0.837 ± 0.007 mm, respectively, with a mean difference of −0.009 mm (97.2% CI −0.028 to 0.011, *p =* 0.309). HbA1c level at 24 mo was significantly lower with sitagliptin than with conventional therapy (6.56% ± 0.05% versus 6.72% ± 0.05%, *p =* 0.008; group mean difference −0.159, 95% CI −0.278 to −0.041). Episodes of serious hypoglycemia were recorded only in the conventional therapy group, and the rate of other adverse events was not different between the two groups. As it was not a placebo-controlled trial and carotid IMT was measured as a surrogate marker of atherosclerosis, there were some limitations of interpretation.

**Conclusions:**

In the PROLOGUE study, there was no evidence that treatment with sitagliptin had an additional effect on the progression of carotid IMT in participants with T2DM beyond that achieved with conventional treatment.

**Trial Registration:**

University Hospital Medical Information Network Clinical Trials Registry UMIN000004490

## Abstract 2

In a randomized controlled trial, Koichi Node and colleagues assess the efficacy of sitagliptin in limiting atherosclerosis among patients over 29 years of age living in Japan.

## Author Summary

**Why Was This Study Done?:**

- Translational research suggests that dipeptidyl peptidase-4 (DPP-4) inhibitors, which are drugs for type 2 diabetes mellitus (T2DM), may prevent cardiovascular disease to a greater extent than other T2DM drugs. However, recently published large clinical trials showed no additional efficacy for DPP-4 inhibitors beyond that expected for the drugs’ glycemic control. Possibly these studies showed no benefit to cardiovascular health because they assessed cardiovascular mortality and other relatively rare outcomes.
- Change in carotid intima-media thickness (IMT) is a simple, noninvasive method to assess atherosclerosis, an intermediate and more common condition that can precede cardiovascular disease.

**What Did the Researchers Do and Find?:**

- We conducted a trial to evaluate whether DPP-4 inhibitors affect atherosclerosis in people with T2DM. In all, 463 participants recruited at 48 medical centers were randomly divided into two groups and treated with sitagliptin, a DPP-4 inhibitor, or other conventional drugs for two years. Carotid IMT was evaluated with ultrasonography in a blinded manner.
- We found that annual changes in carotid IMT were not significantly different between the two groups. On the other hand, the decrease in HbA1c and in the incidence of hypoglycemia with sitagliptin were superior to conventional therapy. The rate of other adverse events was not different between the two groups.

**What Do These Findings Mean?:**

- These findings provide no evidence that sitagliptin slows the progression of carotid intima-media thickening in participants with T2DM to a greater extent than conventional drugs. As some of the conventional anti-T2DM drugs have shown anti-atherosclerotic effects in clinical trials, the present findings do not mean that sitagliptin does not possess anti-atherosclerotic properties in participants with T2DM.
- Importantly, however, sitagliptin is useful for controlling hyperglycemia for clinical setting.

## Introduction

Atherosclerosis, often caused by hypertension, diabetes mellitus, or dyslipidemia, causes ischemic diseases of the brain, heart, and kidney, which increase mortality and morbidity worldwide. Above all, diabetes mellitus is a critical factor in the development of vascular injury. The global prevalence of type 2 diabetes mellitus (T2DM) has been estimated to be 171 million cases, and it is projected to reach double this estimate or more by 2030 [1]. Epidemiological studies have shown that the mortality caused by T2DM is equivalent to that resulting from coronary artery disease (CAD) [2–4].

Noninvasive assessment of carotid artery intima-media thickness (IMT) is widely used to estimate not only carotid but also systemic atherosclerosis, because carotid IMT is independently associated with both the presence of CAD [5–8] and the occurrence of cardiovascular events [9,10], including after adjustment for conventional cardiovascular risk factors. It has also been suggested that an increased carotid IMT is strongly associated with the presence of CAD in people with T2DM [11]. Therefore, evaluation of carotid IMT is helpful when treating people with T2DM.

Dipeptidyl peptidase-4 (DPP-4) inhibitors are antihyperglycemic drugs that stimulate insulin release from pancreatic β-cells by sparing incretin hormones, such as glucagon-like peptide-1 (GLP-1), from degradation by DPP-4. GLP-1 analogues and DPP-4 inhibitors have also been shown to have extra-pancreatic effects beyond glycemic control, including anti-atherosclerotic properties [12,13]. Previous meta-analyses of DPP-4 inhibitors in cardiovascular diseases have demonstrated decreases in cardiovascular events [14,15], while large-scale clinical trials have found that DPP-4 inhibitors neither increased nor decreased the onset of cardiovascular events [16–18]. The SAVOR-TIMI 53 trial reported a significant 27% increase in the incidence of hospitalization for heart failure [16], indicating that it is unclear whether DPP-4 inhibitors have a cardioprotective effect in the clinical setting. Moreover, the anti-atherosclerotic effects of DPP-4 inhibitors are not yet fully clarified. We designed the PROLOGUE (Program of Vascular Evaluation under Glucose Control by DPP-4 Inhibitor) study to investigate the effect of sitagliptin on carotid IMT in people with T2DM.

## Methods

### Study Design and Participants

The rationale and design of the PROLOGUE study have already been described [19]. The study was a multicenter PROBE (prospective, randomized, open label, blinded endpoint) trial carried out in 48 Japanese medical centers. The study protocol and all amendments were reviewed and approved by each center’s ethics committee (S1 and S2 Texts). The study was conducted in accordance with the ethical principles of the Declaration of Helsinki and conformed with good clinical practices and applicable regulatory requirements. All participants gave written consent to participate after having been informed about the nature and purpose of the study, the participation/termination conditions, and the possible risks and benefits of treatment.

We enrolled 463 people with T2DM between November 2010 and September 2012. The inclusion criteria were age ≥ 30 y and presence of T2DM with HbA1c ≥ 6.2% and < 9.4% despite treatment with diet, exercise, and/or conventional antidiabetic agents. The exclusion criteria were as follows: type 1 diabetes mellitus; undergoing insulin treatment; administration of DPP-4 inhibitors and/or GLP-1 analogues before randomization; heart failure with New York Heart Association functional class III or IV; a history of diabetic ketoacidosis or diabetic coma within the 6 mo prior to randomization; a history of myocardial infarction, angina pectoris, percutaneous transluminal coronary angioplasty, or bypass surgery; a history of cerebral infarction, cerebral hemorrhage, subarachnoid hemorrhage, or transient ischemic attack within the 3 mo prior to randomization; serious renal dysfunction (estimated glomerular filtration rate < 30 ml/min/1.73 m^2^ or dialysis); pregnancy or possible pregnancy; lack of informed consent; and judgment of the investigator that an individual is ineligible for inclusion in the study.

Following the initial echocardiographic estimation of carotid IMT, eligible participants were randomly assigned (ratio 1:1) either to receive conventional therapy plus sitagliptin (sitagliptin group) or to continue with only conventional therapy (diet, exercise, and/or antidiabetic agents, except for DPP-4 inhibitors, GLP-1 analogues, and insulin; conventional therapy group). The randomization was done at the PROLOGUE data center using a modified minimization method with a biased-coin assignment balancing on age (<65 or ≥65 y), sex, use of statins, use of antidiabetic agents (nonpharmacological or pharmacological), HbA1c (<7.0% or ≥7.0%), office systolic blood pressure (<135 or ≥135 mm Hg), and maximum IMT (<1.0 or ≥1.0 mm). The random allocation with a stratified technique was generated automatically by a centralized web-based tool that could not be influenced by the researchers. The treatment target level of HbA1c in both groups was <6.2%. All participants were followed annually for 2 y until September 2014 (S1 Fig).

### Measurement of Carotid Intima-Media Thickness

Carotid ultrasound examinations were performed within 1 mo prior to study inclusion, and at 12 and 24 mo after randomization. High-resolution carotid ultrasonography was performed in each ultrasound laboratory in a blinded manner using standardized imaging protocols by expert sonographers trained in the measurement of carotid IMT [20]. All ultrasound systems were equipped with linear transducers of more than 7.5 MHz. Longitudinal B-mode images, perpendicular to the ultrasound beam, with a 4-cm imaging depth, were obtained from the distal common carotid arteries (CCAs), bulbs, and proximal internal carotid arteries (ICA) on both sides. The lateral probe incidence was used to obtain CCA images, using external landmarks with an original semicircular protractor developed for the purpose. The IMT primary parameter measured and calculated was change in the mean far wall CCA IMT in the right and left CCAs 10 mm from the bulb. In addition, the following far wall IMTs were measured: the maximum IMT of the CCA, the mean of the mean IMTs of the CCA, bulb, and ICA, and the mean of the maximum IMTs of the CCA, bulb, and ICA. We used the method recommended by the Mannheim carotid IMT consensus [21]. Plaque was defined as the presence of focal wall thickening at least 50% greater than that of the surrounding vessel wall, or as a focal region with IMT > 1.1 mm protruding into the lumen and distinct from the adjacent boundary. The plaque gray scale median [22] and plaque area far and near to the wall were also measured. If there were multiple carotid plaques in participants, the most echolucent one was selected for measurement of plaque area and gray scale value. The optimized R-wave-gated still frames of the carotid IMT were stored as JPEG files, and all parameters were measured in a core laboratory (University of Tsukuba). A single expert analyst who was unaware of the clinical information of the participants measured all the IMT values using an automatic IMT measurement software program in a blinded manner (Vascular Research Tools 5, Medical Imaging Applications) [23]. The analyst selected the best images of the right and left CCA, bulb, and ICA. In this region, the software identified the lumen/intima and the media/adventitia borders, and calculated the distance between them.

### Endpoints

The primary endpoint was the percentage change in mean CCA IMT at 24 mo after randomization. The secondary endpoints, at 12 and 24 mo, were (1) the mean and maximum IMT values and changes at the CCA, bulb, and ICA (except for the primary endpoint); (2) plaque area and plaque gray scale median; (3) the values and changes in glycemic profiles (HbA1c, fasting glucose level, insulin concentration, 1,5-anhydroglucitol,1,4-anhydro-D-glucitol, HOMA-β, HOMA-R); lipoprotein profiles (total cholesterol, high-density lipoprotein cholesterol, triglyceride, small dense low-density lipoprotein, malondialdehyde-modified low-density lipoprotein, remnant-like particle cholesterol); renal function (creatinine, cystatin C, urinary albumin/creatinine ratio, estimated glomerular filtration rate); high molecular weight adiponectin; physiological parameters (body weight, blood pressure); and (4) adjudicated clinical events and adverse events.

### Statistical Analysis

Based on previous reports, we assumed that the annual change in mean CCA IMT would be −0.005 mm/y in the sitagliptin group and +0.005 mm/y in the conventional therapy group [24,25]. The necessary sample size was calculated on the assumption of a 0.01-mm group difference in the primary endpoint at the final assessment, with a standard deviation (SD) of 0.06 for individual differences and 80% power for a two-sided, two-sample *t*-test at a 0.05 significance level. The target sample size was set at 567 participants per group in anticipation of a 20% dropout rate, with total sample size at 1,200 participants. However, as there has been increasing evidence that DPP-4 inhibitors can reduce HbA1c safely and adequately, they have been quite widely prescribed in Japan. As a consequence, it was difficult to recruit participants who were not taking them, and also to continue the trial without prescribing them. Based on these circumstances, we recalculated the necessary sample size assuming that DPP-4 inhibitors have protective effects similar to those of thiazolidine derivatives, which were reported to prevent carotid intima-media thickening in the CHICAGO study [26]. In the sample size recalculation, we assumed a 0.02-mm group difference with an SD of 0.06 for individual differences and 80% power for a two-sided, two-sample *t*-test at a 0.05 significance level. The target sample size was re-estimated at 238 participants per group in anticipation of a 20% dropout rate, and the total sample size was set at 500 in June 2012. An interim analysis was done for the purpose of investigating futility or overwhelming efficacy of the study and was performed by an independent data monitoring committee at 1 y after the completion of enrollment. The investigators and participants remained blinded to the results of this interim analysis. Significance was evaluated using the Pocock method (with the stopping boundary *p* ≤ 0.0311 at the first interim analysis), resulting in approval of study continuation.

The data for the primary and secondary endpoints were collected at each time point. The statistical analysis and reporting of this trial were conducted in accordance with the CONSORT guidelines, with the primary analyses based on the intention to treat principle (S3 Text). For the baseline variables, summary statistics were constructed employing frequencies and proportions for categorical data and mean and SD for continuous variables. The baseline variables were compared using Fisher’s exact test for categorical outcomes and unpaired *t*-tests for continuous variables, as appropriate.

For the primary analysis comparing the treatment effects, the baseline-adjusted means and their 97.2% CIs, estimated by analysis of covariance (ANCOVA), for the change in average carotid IMT at 24 mo were compared between the treatments (sitagliptin group versus conventional therapy group). This analysis was carried out taking into account the variation caused by treatment effects, and using age, sex, statin use, prerandomization treatment type, baseline HbA1c, baseline office systolic blood pressure, and baseline maximum IMT as covariates. The significance level of the final analysis was set at 0.0277 (two-sided). We did not impute missing observations for the primary analysis, but the mixed-effects model for repeated measures was applied as a sensitivity analysis to examine the effect of missing data. The secondary analyses were performed in the same manner as the primary analysis. The baseline-adjusted means of the secondary endpoints at each time point were estimated by ANCOVA with treatment effects, age, sex, statin usage, pre-randomization treatment type, baseline HbA1c, baseline office systolic blood pressure, and the baseline value of the secondary efficacy parameter as covariates.

All comparisons were planned, and all *p*-values were two-sided. A *p*-value < 0.05 was considered statistically significant. All statistical analyses were performed using SAS, version 9.4 (SAS Institute).

### Study Management

The organization of the study is detailed in S4 Text. A steering committee was responsible for the study design and scientific execution. An independent efficacy and safety evaluation committee, consisting of six members blinded to any information related to group allocation, evaluated each clinical and adverse event. The independent data monitoring committee, composed of three members, independently reviewed the interim analysis to advise whether it was appropriate to continue the study. An independent audit team inspected several main medical centers to ensure the quality of the data. All medical centers participating in this study are listed in S4 Text. Changes in the protocol, including definition of the primary endpoint, change of sample size, and enforcement of interim analysis, are described in S2 Text.

Data for this study were deposited in the Dryad Digital Repository [27].

## Results

### Study Population

**Fig 1:**
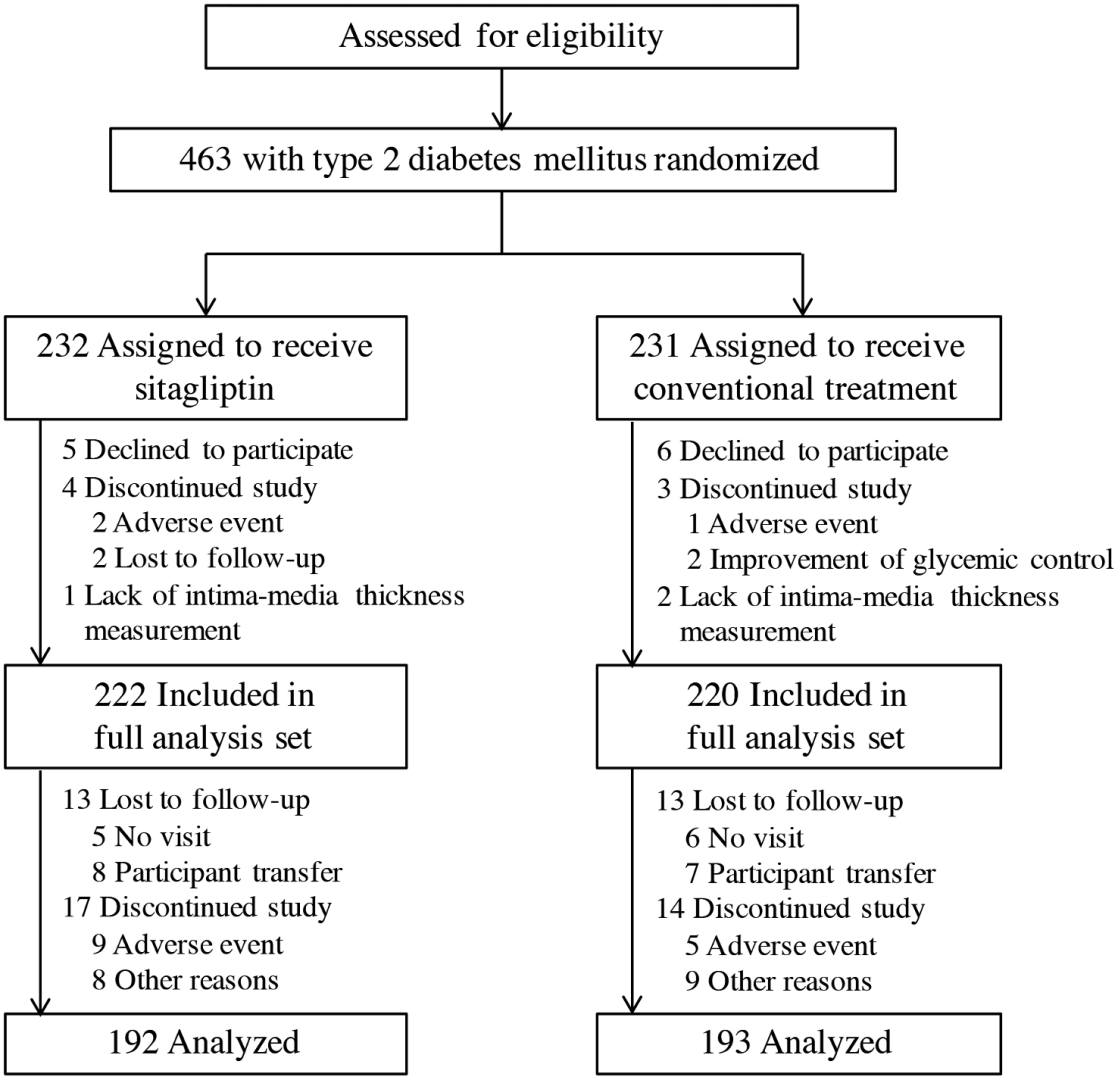
Participant disposition.

In all, 463 people with T2DM were enrolled and randomly assigned to the two groups (232 and 231 to the sitagliptin and conventional therapy groups, respectively). We obtained complete endpoint information at the end of the study for 222 participants in the sitagliptin group and 220 participants in the conventional therapy group (Fig 1). There were no significant differences in baseline characteristics between the two groups (Table 1).

**Table 1:** Baseline demographics and clinical characteristics.

Characteristic	Sitagliptin Group (*n =* 222)	Conventional Therapy Group (*n =* 220)	*p*-Value
Age, years	69.2 ± 9.3	69.5 ± 9.2	0.708
Male	146 (65.8%)	151 (68.6%)	0.544
Body mass index, kg/m^2^	25.3 ± 4.1	24.9 ± 4.0	0.268
Hypertension	181 (81.5%)	166 (75.5%)	0.133
Dyslipidemia	163 (73.4%)	148 (67.3%)	0.176
Myocardial infarction	46 (20.7%)	55 (25.0%)	0.309
Percutaneous coronary intervention	58 (26.1%)	69 (31.4%)	0.248
Coronary artery bypass grafting	19 (8.6%)	16 (7.3%)	0.725
Chronic heart failure	15 (6.8%)	26 (11.8%)	0.073
Arrhythmia	32 (14.4%)	32 (14.5%)	1.000
Stroke	27 (12.2%)	30 (13.6%)	0.672
Systolic blood pressure, mm Hg	130.0 ± 15.7	128.8 ± 16.5	0.403
Diastolic blood pressure, mm Hg	72.8 ± 10.7	71.7 ± 11.5	0.278
HbA1c, percent	6.96 ± 0.64	6.96 ± 0.55	0.974
Fasting plasma glucose, mmol/l	7.67 ± 2.31	7.49 ± 2.05	0.396
Low-density lipoprotein cholesterol, mmol/l	2.45 ± 0.67	2.41 ± 0.73	0.602
Serum creatinine, μmol/l	75.5 ± 20.8	76.3 ± 23.0	0.683
Estimated glomerular filtration rate, ml/min/1.73 m^2^	66.5 ± 17.4	66.8 ± 18.1	0.830
Mean CCA IMT, mm	0.829 ± 0.166	0.835 ± 0.190	0.725
Mean bulb IMT, mm	1.109 ± 0.469	1.114 ± 0.421	0.912
Mean ICA IMT, mm	0.775 ± 0.340	0.795 ± 0.316	0.557
Maximum CCA IMT, mm	1.052 ± 0.228	1.076 ± 0.264	0.298
Maximum bulb IMT, mm	1.583 ± 0.686	1.601 ± 0.635	0.787
Maximum ICA IMT, mm	1.039 ± 0.440	1.081 ± 0.448	0.360
Plaque area, mm^2^	11.36 ± 7.41	11.72 ± 9.24	0.724
Plaque gray scale median	50.9 ± 22.5	52.5 ± 21.6	0.568

Data are presented as number (percent) or mean (SD).

### Carotid Intima-Media Thickness

At baseline, the mean CCA IMT (± SD) was 0.829 ± 0.166 mm in the sitagliptin group and 0.835 ± 0.190 mm in the conventional therapy group (Table 1). The baseline-adjusted mean (± standard error [SE]) CCA IMT at 24 mo in the sitagliptin and conventional therapy groups was 0.827 ± 0.007 mm and 0.837 ± 0.007 mm, respectively, with a mean difference of −0.009 mm (97.2% CI −0.028 to 0.011, *p =* 0.309). Although the change in maximum ICA IMT at 24 mo was significantly greater in the sitagliptin group (*p =* 0.038), the values of mean bulb IMT, mean ICA IMT, maximum CCA IMT, maximum bulb IMT, plaque area, and plaque gray scale median at 24 mo did not differ significantly between the two groups at any time point (Table 2).

**Table 2:** IMT measurement, plaque area, and plaque gray scale median at each time point.

Endpoint	Time Point	Baseline-Adjusted Mean ± SE		Group Difference in Baseline-Adjusted Mean (95% CI)	*p*-Value
		Sitagliptin Group (*n =* 222)	Conventional Therapy Group (*n =* 220)		
Mean CCA IMT, mm	12 mo	0.818 ± 0.007	0.822 ± 0.007	−0.004 (−0.022 to 0.013)	0.628
	24 mo	0.827 ± 0.007	0.837 ± 0.007	−0.009 (−0.028 to 0.011)*	0.309
Mean bulb IMT, mm	12 mo	1.146 ± 0.032	1.169 ± 0.032	−0.023 (−0.103 to 0.056)	0.569
	24 mo	1.164 ± 0.034	1.181 ± 0.032	−0.016 (−0.100 to 0.066)	0.691
Mean ICA IMT, mm	12 mo	0.882 ± 0.033	0.909 ± 0.032	−0.026 (−0.107 to 0.054)	0.521
	24 mo	0.764 ± 0.025	0.824 ± 0.024	−0.059 (−0.122 to 0.002)	0.060
Maximum CCA IMT, mm	12 mo	1.048 ± 0.011	1.039 ± 0.011	0.008 (−0.019 to 0.037)	0.551
	24 mo	1.066 ± 0.011	1.059 ± 0.011	0.007 (−0.022 to 0.036)	0.635
Maximum bulb IMT, mm	12 mo	1.566 ± 0.044	1.589 ± 0.043	−0.022 (−0.130 to 0.086)	0.686
	24 mo	1.659 ± 0.046	1.673 ± 0.044	−0.014 (−0.126 to 0.098)	0.802
Maximum ICA IMT, mm	12 mo	1.178 ± 0.044	1.210 ± 0.043	−0.031 (−0.139 to 0.076)	0.564
	24 mo	1.049 ± 0.341	1.138 ± 0.032	−0.089 (−0.173 to −0.004)	0.038
Plaque area, mm^2^	12 mo	12.94 ± 0.78	12.49 ± 0.70	0.45 (−1.37 to 2.26)	0.626
	24 mo	12.06 ± 0.58	10.80 ± 0.52	1.26 (−0.12 to 2.63)	0.073
Plaque gray scale median	12 mo	60.48 ± 4.34	61.05 ± 3.83	−0.58 (−10.61 to 9.46)	0.909
	24 mo	48.86 ± 2.43	52.28 ± 2.18	−3.42 (−9.17 to 2.33)	0.242

Values were adjusted for the baseline values using ANCOVA.

*97.2% CI.

The effect of sitagliptin on carotid IMT in older participants (≥70 y) was evaluated (S1 Table). However, there was no statistically significant difference in effect between the older and younger participants. Accordingly, the results did not depend on the age of the participants.

### Glucose Metabolism and Other Parameters

The baseline levels of HbA1c were 6.96% ± 0.64% and 6.96% ± 0.55% in the sitagliptin and conventional therapy groups, respectively (mean ± SD; Table 1). The baseline-adjusted mean (± SE) at each time point and the change in HbA1c level are shown in Tables S2 and 3. HbA1c level decreased to 6.56% ± 0.04% in the sitagliptin group and to 6.67% ± 0.04% in the conventional therapy group at 12 mo (*p =* 0.047), and to 6.56% ± 0.05% in the sitagliptin group and 6.72% ± 0.05% in the conventional therapy group at 24 mo (*p =* 0.008). While the change in 1,5-anhydroglucitol,1,4-anhydro-D-glucitol at 24 mo in the conventional therapy group was significantly greater than that in the sitagliptin group (*p =* 0.048), there was no significant difference between the groups in fasting plasma glucose at 24 mo (*p =* 0.787). There were no significant differences between the two groups in changes in the other parameters, including body weight, blood pressure, renal function, and metabolic and lipid profiles.

**Table 3:** Physiological and biochemical parameters at 24 mo.

Parameter	Baseline-Adjusted Mean ± SE		Group Difference in Baseline-Adjusted Mean (95% CI)	*p*-Value
	Sitagliptin Group (*n =* 222)	Conventional Therapy Group (*n =* 220)		
Body weight (kg)	64.3 ± 0.3	64.9 ± 0.3	−0.598 (−1.293 to 0.095)	0.090
Body mass index (kg/m^2^)	24.9 ± 0.1	25.1 ± 0.1	−0.245 (−0.519 to 0.029)	0.079
Waist circumference (cm)	89.5 ± 0.5	89.8 ± 0.5	−0.330 (−1.681 to 1.021)	0.630
Systolic blood pressure (mm Hg)	129.7 ± 1.2	129.9 ± 1.2	−0.236 (−3.304 to 2.832)	0.879
Diastolic blood pressure (mm Hg)	72.7 ± 0.8	72.1 ± 0.8	0.582 (−1.409 to 2.574)	0.565
Pulse rate (bpm)	71.9 ± 0.9	70.5 ± 0.8	1.418 (−0.694 to 3.531)	0.187
HbA1c (percent)	6.56 ± 0.05	6.72 ± 0.05	−0.159 (−0.278 to −0.041)	0.008
Fasting plasma glucose (mmol/l)	7.09 ± 0.15	7.14 ± 0.14	−0.051 (−0.429 to 0.325)	0.787
HOMA-β (percent)	90.0 ± 11.0	92.5 ± 10.2	−2.500 (−29.21 to 24.21)	0.853
HOMA-R	5.05 ± 0.72	4.65 ± 0.66	0.408 (−1.327 to 2.144)	0.643
Insulin concentration (pmol/l)	94.4 ± 11.1	94.0 ± 10.3	0.387 (−26.57 to 27.35)	0.977
1,5-anhydroglucitol,1,4-anhydro-D-glucitol (μmol/l)	103.5 ± 2.9	96.3 ± 2.8	7.200 (0.047 to 14.35)	0.048
High molecular weight adiponectin (μmol/l)	0.16 ± 0.01	0.19 ± 0.01	−0.022 (−0.046 to 0.002)	0.070
Low-density lipoprotein cholesterol (mmol/l)	2.42 ± 0.05	2.50 ± 0.05	−0.077 (−0.199 to 0.046)	0.219
High-density lipoprotein cholesterol (mmol/l)	1.35 ± 0.01	1.37 ± 0.01	−0.019 (−0.063 to 0.025)	0.391
Triglyceride (mmol/l)	1.54 ± 0.06	1.48 ± 0.06	0.063 (−0.095 to 0.222)	0.433
Small dense low-density lipoprotein cholesterol (mmol/l)	0.86 ± 0.03	0.92 ± 0.03	−0.066 (−0.153 to 0.020)	0.132
Remnant-like particle cholesterol (mmol/l)	0.12 ± 0.01	0.13 ± 0.01	−0.005 (−0.027 to 0.017)	0.662
Malondialdehyde-modified low-density lipoprotein (U/l)	115.4 ± 2.81	121.1 ± 2.79	−5.645 (−12.66 to 1.374)	0.114
Aspartate aminotransferase (U/l)	26.6 ± 0.8	25.9 ± 0.8	0.636 (−1.441 to 2.714)	0.547
Alanine aminotransferase (U/l)	23.8 ± 0.9	23.7 ± 0.9	0.117 (−2.137 to 2.372)	0.918
Lactic dehydrogenase (U/l)	205.2 ± 2.1	205.7 ± 2.0	−0.573 (−5.867 to 4.719)	0.831
Amylase (U/l)	82.1 ± 3.1	75.9 ± 3.1	6.078 (−2.059 to 14.21)	0.141
Blood urea nitrogen (mmol/l)	6.28 ± 0.13	6.46 ± 0.13	−0.178 (−0.510 to 0.152)	0.290
Serum creatinine (μmol/l)	79.4 ± 1.1	78.3 ± 1.1	1.082 (−1.756 to 3.920)	0.454
Estimated glomerular filtration rate (ml/min/1.73 m^2^)	64.1 ± 0.8	64.7 ± 0.7	−0.607 (−2.503 to 1.289)	0.529
Cystatin C (μmol/l)	0.081 ± 0.001	0.082 ± 0.001	−0.0006 (−0.005 to 0.004)	0.800
Urinary albumin/creatinine ratio (mg/g)	105.6 ± 19.3	70.1 ± 18.9	35.59 (−12.05 to 83.23)	0.142
Uric acid (μmol/l)	343.6 ± 4.9	343.1 ± 4.9	0.557 (−11.7 to 12.8)	0.929

Values were adjusted for the baseline values using ANCOVA.

In the conventional therapy group, the additional use of a sulfonylurea, biguanide, α-glucosidase inhibitor, or thiazolidinedione for the management of glycemic control increased during the 24-mo observation period. Conversely, the usage of other antidiabetic agents, except for biguanide drugs, did not increase in the sitagliptin group (Table 4). The average dose of sitagliptin was 48 ± 8 mg at baseline, 62 ± 24 mg at 12 mo, and 64 ± 26 mg at 24 mo (S3 Table).

**Table 4:** Antidiabetic and other agents during the study.

Agent	Baseline			12 mo Follow-Up			24 mo Follow-Up		
	Sitagliptin Group (*n =* 222)	Conventional Therapy Group (*n =* 220)	*p*-Value	Sitagliptin Group (*n =* 204)	Conventional Therapy Group (*n =* 201)	*p*-Value	Sitagliptin Group (*n =* 192)	Conventional Therapy Group (*n =* 193)	*p*-Value
Sulfonylurea	56 (25.2%)	52 (23.6%)	0.740	40 (19.6%)	66 (32.8%)	0.003	36 (18.8%)	61 (31.6%)	0.005
Biguanide	34 (15.3%)	32 (14.5%)	0.894	40 (19.6%)	69 (34.3%)	0.001	45 (23.4%)	68 (35.2%)	0.014
α-Glucosidase inhibitor	72 (32.4%)	66 (30.0%)	0.609	54 (26.5%)	84 (41.8%)	0.001	46 (24.0%)	81 (42.0%)	<0.001
Thiazolidinedione	53 (23.9%)	53 (24.1%)	1.000	40 (19.6%)	64 (31.8%)	0.006	37 (19.3%)	62 (32.1%)	0.005
Glinide	7 (3.2%)	19 (8.6%)	0.015	4 (2.0%)	25 (12.4%)	<0.001	3 (1.6%)	21 (10.9%)	<0.001
Statin	169 (76.1%)	162 (73.6%)	0.584	151 (74.0%)	143 (71.1%)	0.578	142 (74.0%)	135 (69.9%)	0.427
Fibrate	3 (1.4%)	3 (1.4%)	1.000	3 (1.5%)	3 (1.5%)	1.000	3 (1.6%)	3 (1.6%)	1.000
Angiotensin II receptor blocker	132 (59.5%)	113 (51.4%)	0.104	120 (58.8%)	105 (52.2%)	0.195	117 (60.9%)	100 (51.8%)	0.081
Angiotensin-converting enzyme inhibitor	26 (11.7%)	36 (16.4%)	0.172	23 (11.3%)	32 (15.9%)	0.193	19 (9.9%)	31 (16.1%)	0.095

Data are presented as the number of participants (percent).

In each participant, adherence to medications was monitored by counting the residual number of tablets at each clinic visit. If the average daily consumption of all tablets combined in a participant was less than 75% of that prescribed, the participant was defined as having “poor adherence” at the end of the study. There were seven such participants in the sitagliptin group, and nine in the conventional therapy group.

### Adjudicated Clinical and Adverse Events

During the 24 mo of follow-up, five participants died, three in the sitagliptin group and two in the conventional therapy group (nontuberculous mycobacterial infection, multiple organ failure following bone fracture, coma of unknown cause, or sudden death); sudden death occurred in one participant in each group. Nonfatal myocardial infarction occurred in two participants in the sitagliptin group and in one in the conventional therapy group. Nonfatal stroke occurred in one participant in each group. Two participants in each group initiated insulin therapy. Deterioration of renal function, defined as a greater than 2-fold increase in serum creatinine level, was seen in three participants in the sitagliptin group and in one in the conventional therapy group. Hypoglycemia, which is one of the most clinically important adverse events, was recorded only in the conventional therapy group. Heart failure occurred in two participants in the sitagliptin group and in four in the conventional therapy group. Acute pancreatitis and pancreatic cancer were not observed in either group (Table 5).

**Table 5:** Summary of adjudicated clinical events and adverse events.

Event	Sitagliptin Group (*n =* 222)	Conventional Therapy Group (*n =* 220)
**Death**	3	2
**Adjudicated clinical events**	9	6
Sudden death	1	1
Nonfatal myocardial infarction	2	1
Nonfatal stroke	1	1
Deterioration of renal function	3	1
Transfer to insulin therapy	2	2
**Adverse events**	32	29
Hypoglycemia	0	7
Cardiovascular disease	12	7
*Heart failure*	2	4
*Angina pectoris*	6	3
*Peripheral arterial disease*	1	0
*Arrhythmia*	1	0
*Other cardiovascular disorders*	2	0
Edema	2	1
Skin eruption	3	0
Gastrointestinal disorders	6	3
Cancer	0	6
Others	9	5

Data are presented as the number of participants.

## Discussion

### Main Findings

To our knowledge, the PROLOGUE study is the largest trial to investigate whether DPP-4 inhibitors slow the progression of carotid IMT in participants with T2DM. The major finding of the study is that the serial change in mean CCA IMT was not significantly different between conventional treatment plus sitagliptin and conventional treatment alone. The change in HbA1c was significantly greater in the sitagliptin group. These results suggest that sitagliptin failed to inhibit IMT progression relative to conventional therapy despite its glucose-lowering effect.

### Intima-Media Thickness in People with Type 2 Diabetes Mellitus

The primary endpoint of carotid IMT in this study, which can be evaluated noninvasively using ultrasonography, reflects atherosclerotic status and has been used to predict the risk of myocardial infarction and stroke [28]. A positive association exists between carotid IMT and subsequent cardiovascular events in the general population, independent of other major risk factors [29]. A single measurement of carotid IMT and assessment of carotid plaque increases the predictive power of cardiovascular disease risk assessment [30–35]. Accordingly, the American College of Cardiology Foundation/American Heart Association guidelines [36] give carotid IMT and plaque measurement for cardiovascular risk stratification as a class IIA recommendation. Repeated carotid IMT measurements are an accepted approach to test the effects of interventions on carotid IMT progression. The Multi-Ethnic Study of Atherosclerosis [31] showed a positive association between carotid IMT progression and stroke incidence. Although two meta-regression analyses of clinical trial data [37,38] reported conflicting results on the relationship between carotid IMT progression and cardiovascular events, many reports have supported its use in pathophysiological studies and clinical trials, such that the perception of carotid IMT has shifted from that of a secondary endpoint to that of a surrogate marker of the risk of cardiovascular events for evaluating therapeutic interventions in atherosclerotic disease [39,40]. It was on this basis that we undertook the PROLOGUE study to determine whether treatment with a DPP-4 inhibitor, sitagliptin, is more effective than conventional therapy at inhibiting atherosclerotic progression in Japanese people, using carotid IMT as a surrogate marker. Measurement of carotid IMT in a blinded manner and in a core laboratory—as done in this study—avoids bias, improves accuracy, enhances the credibility of image assessments, and ensures consistency. A highly standardized imaging acquisition protocol in a fully blinded manner is recommended [41].

In the CHICAGO (Carotid Intima-Media Thickness in Atherosclerosis Using Pioglitazone) trial, the inhibitory effect of 15–45 mg/d of pioglitazone on mean CCA IMT progression was evident during 18 mo of observation, in comparison with 1–4 mg/d glimepiride [26]. Other investigators also demonstrated that pioglitazone slowed the progression of carotid IMT compared with glimepiride [35,42]. In addition to pioglitazone, a biguanide that has been suggested to inhibit DPP-4 activity and increase GLP-1 concentration [43,44] also reduced the progression of carotid IMT [45,46]. In studies of small numbers of people, DPP-4 inhibitors including sitagliptin were reported to inhibit the progression of carotid IMT [47,48]. The SPEAD-A study revealed that treatment with alogliptin, the other DPP-4 inhibitor, attenuated the progression of carotid IMT compared with conventional treatment during 24 mo of follow-up [49]. More recently, the SPIKE trial demonstrated that sitagliptin attenuated carotid IMT in people with T2DM who were under treatment with insulin [50]. However, compared with these studies showing positive results, in the PROLOGUE study the change in mean CCA IMT in the sitagliptin group did not differ significantly from that in the conventional therapy group. Participants on insulin treatment were excluded from the present study, HbA1c values at baseline were lower (PROLOGUE study: 6.96% [sitagliptin], 6.96% [conventional]; SPEAD-A study: 7.3% [alogliptin], 7.2% [conventional]; SPIKE trial: 8.1% [sitagliptin], 8.0% [conventional]), and the difference between the treatment groups in the change in HbA1c over 24 mo was smaller (PROLOGUE study: 0.14%; SPEAD-A study: 0.2%; SPIKE trial: 0.3%). In addition, the prevalence of statin use was higher in the PROLOGUE study than in the SPEAD-A study and the SPIKE trial (PROLOGUE study: 76.1% [sitagliptin], 73.6% [conventional]; SPEAD-A study: 38% [alogliptin], 46% [conventional]; SPIKE trial: 48% [sitagliptin], 46% [conventional]) [49,50]. Thus, it may not be appropriate to directly compare these different results, as the baseline characteristics of the participants, such as severity of T2DM, HbA1c level, and carotid IMT, differed between them. In the PROLOGUE study, even in the conventional therapy group mean CCA IMT had increased only slightly at 24 mo, which seems to be lower than values reported previously. It has been reported that the annual progression of carotid IMT in Japanese people with T2DM is 0.016 to 0.035 mm [25,51] even with aggressive antidiabetic treatment. In the CHICAGO study, the glimepiride arm showed a 0.012-mm increase in mean CCA IMT during 18 mo of follow-up. Interestingly, in the PROLOGUE study additional use of both pioglitazone and biguanides increased in the conventional therapy group, possibly contributing to the suppression of IMT progression. Additionally, both the sitagliptin and conventional treatment groups achieved strict overall management of risk factors, including glycemic control, although the reduction of HbA1c was greater in the sitagliptin group than in the conventional therapy group. On these grounds, the progression of carotid IMT might be inhibited in both groups, in contrast to previous reports [25,26,47–51].

### DPP-4 Inhibitors and Cardiovascular Disease

There are published experimental data showing that incretin therapy, including treatment with GLP-1 analogues or DPP-4 inhibitors, attenuates atherosclerosis. GLP-1 and its analogues inhibit the inflammatory and atherogenic pathways induced by exogenous stimulation with oxidative stress and/or inflammation in vitro [52–55]. In vivo studies have revealed that the administration of GLP-1 analogues or DPP-4 inhibitors reduces high-fat-diet-induced atherosclerosis and stabilizes plaques in apoE knockout mice [55–57]. In the clinical setting, several investigators have demonstrated that GLP-1 analogues or DPP-4 inhibitors improve endothelial function in people with T2DM [58,59]. These lines of evidence suggest that sitagliptin may have beneficial effects on atherosclerosis in humans. Large-scale randomized clinical trials found that DPP-4 inhibitors were safe and effective in the treatment of T2DM. However, they did not reduce cardiovascular events in participants with T2DM who were at high risk for cardiovascular disease or who had a recent onset of acute coronary syndrome [16–18]. In the TECOS trial [18], sitagliptin had no effect on cardiovascular events in participants with T2DM. The discrepancy between the experimental data and recent clinical results requires explanation. One possibility is that the period of observation in clinical studies may have been too short to evaluate the clinical effects of sitagliptin. Efficacy against cardiovascular events can be demonstrated over several years, according to the VADT study and the UK Prospective Diabetes Study. The TECOS trial was designed to show noninferiority for cardiovascular events with sitagliptin. Therefore, we had to clarify the precise effects of sitagliptin on atherosclerosis directly to evaluate carotid IMT. In the PROLOGUE study, intima-media thickening did not differ significantly between the sitagliptin and conventional therapy groups during 24 mo of follow-up.

In experimental research in patients with T2DM, comparisons of drugs are relatively simple and their doses are sometimes much greater than those used in clinical practice. Usually people are also taking other drugs, including antidiabetic, antihypertensive, or lipid-lowering drugs, and it is difficult to exclude their influence. Indeed, the use of thiazolidinediones and biguanides increased as co-therapies in the conventional therapy group in our study (Table 4), which may have diminished the effect of the DPP-4 inhibitor. Moreover, better glycemic control can now be achieved compared with several years ago. This may be the reason why carotid IMT in the conventional therapy group did not increase as much as anticipated. We emphasize that the true purpose of the present study was not to elucidate whether sitagliptin can prevent carotid IMT compared with a placebo, but to investigate whether sitagliptin can confer an additional beneficial effect on carotid IMT in clinical practice.

### Study Limitations

The PROLOGUE study has a few limitations. First, it was conducted with a PROBE design, which might have introduced bias in the assessment of outcomes. Although the primary endpoint was measured by a single observer in a core laboratory who was blinded to the treatment assignments, open-label treatments might affect physicians’ choices of therapy. Second, baseline HbA1c level and carotid IMT were not very high—probably because participants’ diabetes was well-controlled without insulin treatment—in comparison with previous clinical studies that assessed the additional effects of sitagliptin on carotid atherosclerosis relative to conventional antidiabetic drugs [50].

In conclusion, the PROLOGUE study did not provide evidence that treatment with sitagliptin has an additional effect on the progression of carotid IMT in participants with T2DM beyond that achieved with conventional treatment.

## Supporting Information

S1 Fig Study protocol. Inclusion criteria: age ≥ 30 y, T2DM with HbA1c ≥ 6.2% and < 9.4%. Exclusion criteria: type 1 diabetes mellitus; receiving insulin therapy; administration of DPP-4 inhibitors or GLP-1 analogues before randomization; heart failure graded at New York Heart Association functional class III or IV; a history of diabetic ketoacidosis or diabetic coma within the 6 mo prior to randomization; a history of myocardial infarction, angina pectoris, percutaneous transluminal coronary angioplasty, or bypass surgery; a history of cerebral infarction, cerebral hemorrhage, subarachnoid hemorrhage, or transient ischemic attack within the 3 mo prior to randomization, serious renal dysfunction (estimated glomerular filtration rate < 30 ml/min/1.73 m^2^ or dialysis); pregnancy or possible pregnancy; lack of informed consent; and judgment of the investigator that an individual is ineligible for inclusion in the study.
(TIF)

S1 Table The effect of sitagliptin on carotid IMT in younger (<70 y old) and older (≥70 y old) participants. (DOCX)

S2 Table Physiological and biochemical parameters at 12 mo. (DOCX)

S3 Table Doses of sitagliptin during the study, Data are presented as the number of participants (percent). (DOCX)

S1 Text Clinical trial protocol. (DOCX)

S2 Text Study protocol amendment. (DOC)

S3 Text CONSORT statement. (DOC)

S4 Text Organization of PROLOGUE study. (DOCX)

S5 Text Ethical approval document. (JPG)

S6 Text Full competing interests statement. (DOCX)

## Abbreviations

ANCOVA: analysis of covariance
CAD: coronary artery disease
CCA: common carotid artery
ICA: internal carotid artery
IMT: intima-media thickness
SD: standard deviation
SE: standard error
T2DM: type 2 diabetes mellitus
