# Supplementary figures and images for: The Effect of Sitagliptin on Carotid Artery Atherosclerosis in Type 2 Diabetes: The PROLOGUE Randomized Controlled Trial
Source: PLoS Med. 2016 Jun 28;13(6):e1002051. doi: 10.1371/journal.pmed.1002051 (PMC4924847; doi:10.1371/journal.pmed.1002051)

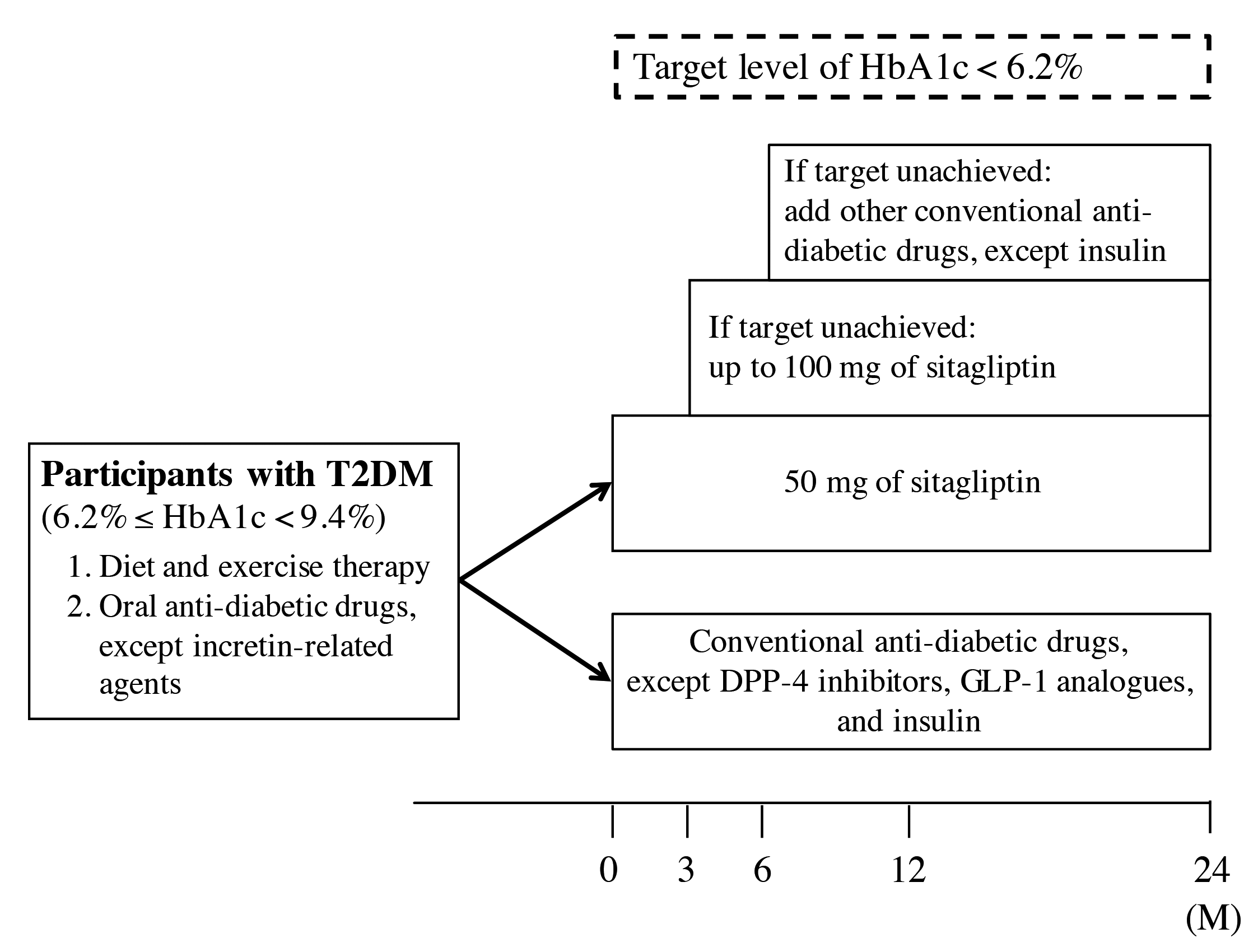

Supplement: S1 Fig — Inclusion criteria: age ≥ 30 y, T2DM with HbA1c ≥ 6.2% and < 9.4%. Exclusion criteria: type 1 diabetes mellitus; receiving insulin therapy; administration of DPP-4 inhibitors or GLP-1 analogues before randomization; heart failure graded at New York Heart Association functional class III or IV; a history of diabetic ketoacidosis or diabetic coma within the 6 mo prior to randomization; a history of myocardial infarction, angina pectoris, percutaneous transluminal coronary angioplasty, or bypass surgery; a history of cerebral infarction, cerebral hemorrhage, subarachnoid hemorrhage, or transient ischemic attack within the 3 mo prior to randomization, serious renal dysfunction (estimated glomerular filtration rate < 30 ml/min/1.73 m2 or dialysis); pregnancy or possible pregnancy; lack of informed consent; and judgment of the investigator that an individual is ineligible for inclusion in the study. (TIF) [file pmed.1002051.s001.TIF]

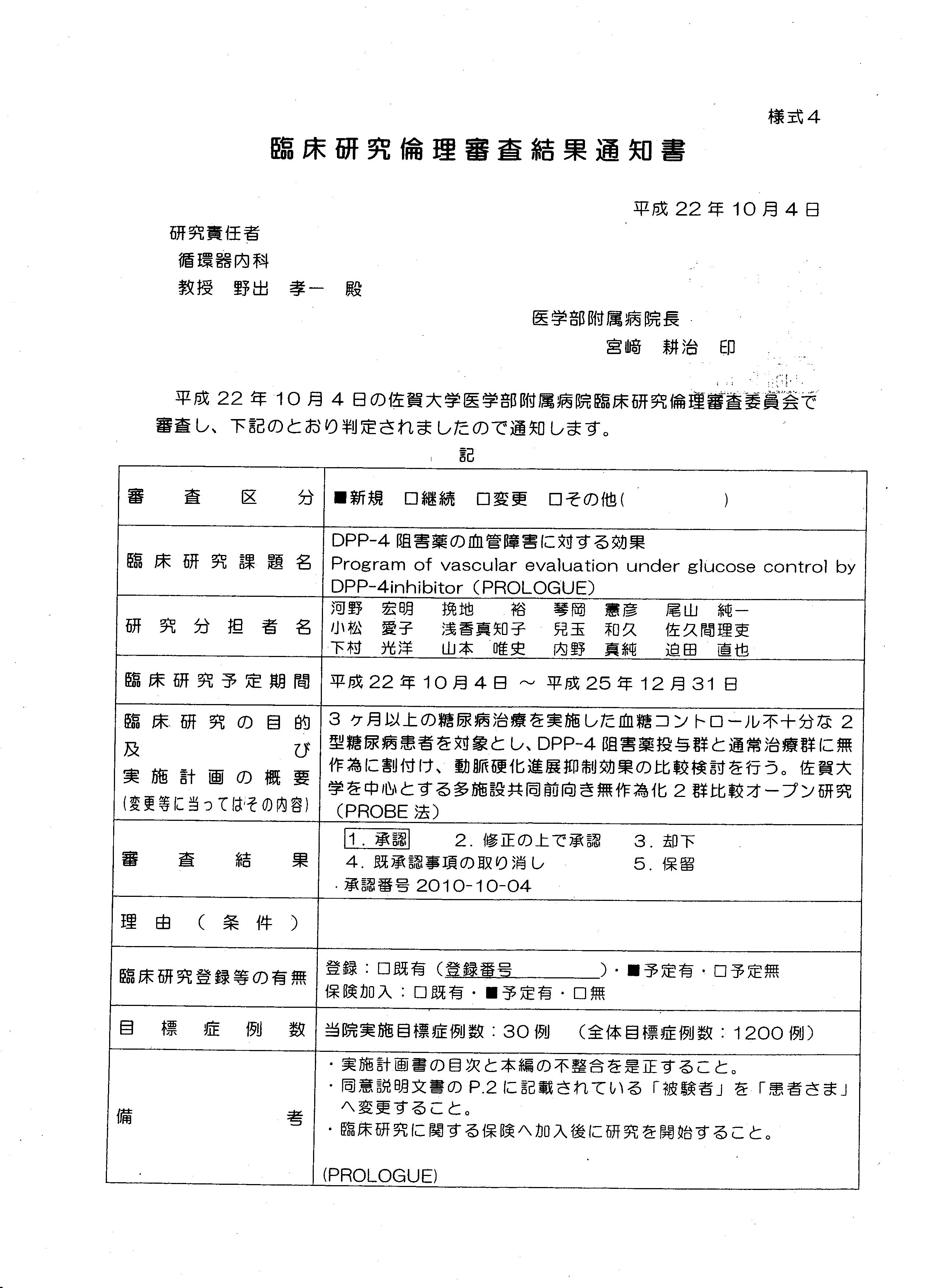

Supplement: S5 Text — (JPG) [file pmed.1002051.s009.jpg]
