# Supplementary material for: The Effect of Sitagliptin on Carotid Artery Atherosclerosis in Type 2 Diabetes: The PROLOGUE Randomized Controlled Trial
Source: PLoS Med. 2016 Jun 28;13(6):e1002051. doi: 10.1371/journal.pmed.1002051 (PMC4924847; doi:10.1371/journal.pmed.1002051)
Supplement: S1 Table — (DOCX) [file pmed.1002051.s002.docx]

**S1 Table. The Effect of sitagliptin on Carotid IMT in younger (<70 years old) and older participants (**≥ **70 years old)**

|  |  | Age < 70 | | | | Age ≥ 70 | | | |
| --- | --- | --- | --- | --- | --- | --- | --- | --- | --- |
|  |  | Sitagliptin  (n = 107)  Baseline-adjusted means ± SE | Conventional  (n = 103)  Baseline-adjusted means ± SE | Group Difference  Mean (95%CI) | *p*-Value | Sitagliptin  (n = 115)  Baseline-adjusted means ± SE | Conventional  (n = 117)  Baseline-adjusted means ± SE | Group Difference  Mean (95%CI) | *p*-Value |
| Mean common carotid artery IMT, mm | 12M | 0.799 ± 0.009 | 0.804 ± 0.009 | -0.005  (-0.029 - 0.018) | 0.672 | 0.833 ± 0.010 | 0.838 ± 0.011 | -0.004  (-0.031 - 0.022) | 0.752 |
|  | 24M | 0.797 ± 0.008 | 0.813 ± 0.008 | -0.015  (-0.036 - 0.005) | 0.144 | 0.851 ± 0.010 | 0.853 ± 0.011 | -0.003  (-0.030 - 0.024) | 0.841 |
| Mean bulb IMT, mm | 12M | 1.054 ± 0.039 | 1.060 ± 0.038 | -0.006  (-0.104 - 0.092) | 0.902 | 1.252 ± 0.054 | 1.280 ± 0.055 | -0.028  (-0.156 - 0.099) | 0.660 |
|  | 24M | 1.088 ± 0.038 | 1.115 ± 0.036 | -0.026  (-0.122 - 0.068) | 0.578 | 1.241 ± 0.055 | 1.237 ± 0.055 | 0.004  (-0.130 - 0.138) | 0.950 |
| Mean internal carotid artery IMT, mm | 12M | 0.806 ± 0.036 | 0.790 ± 0.035 | 0.015  (-0.075 - 0.106) | 0.732 | 0.973 ± 0.056 | 1.043 ± 0.057 | -0.070  (-0.205 - 0.063) | 0.298 |
|  | 24M | 0.710 ± 0.027 | 0.765 ± 0.025 | -0.054  (-0.121 - 0.011) | 0.104 | 0.825 ± 0.041 | 0.886 ± 0.041 | -0.060  (-0.164 - 0.042) | 0.247 |
| Max common carotid artery IMT, mm | 12M | 1.015 ± 0.015 | 0.997 ± 0.014 | 0.017  (-0.019 - 0.054) | 0.348 | 1.076 ± 0.017 | 1.081 ± 0.018 | -0.004  (-0.048 - 0.039) | 0.841 |
|  | 24M | 1.021 ± 0.015 | 1.019 ± 0.015 | 0.002  (-0.036 - 0.041) | 0.904 | 1.106 ± 0.017 | 1.095 ± 0.018 | 0.011  (-0.033 - 0.055) | 0.620 |
| Max bulb IMT, mm | 12M | 1.424 ± 0.055 | 1.408 ± 0.054 | 0.015  (-0.122 - 0.154) | 0.825 | 1.722 ± 0.071 | 1.774 ± 0.074 | -0.052  (-0.221 - 0.116) | 0.540 |
|  | 24M | 1.552 ± 0.053 | 1.552 ± 0.051 | 0.0001  (-0.132 - 0.132) | 0.997 | 1.764 ± 0.073 | 1.784 ± 0.073 | -0.020  (-0.200 - 0.159) | 0.825 |
| Max internal carotid artery IMT, mm | 12M | 1.082 ± 0.052 | 1.064 ± 0.051 | 0.018  (-0.112 - 0.148) | 0.785 | 1.291 ± 0.072 | 1.374 ± 0.074 | -0.082  (-0.255 - 0.089) | 0.344 |
|  | 24M | 0.971 ± 0.036 | 1.044 ± 0.033 | -0.073  (-0.164 - 0.016) | 0.109 | 1.137 ± 0.055 | 1.243 ± 0.055 | -0.105  (-0.244 - 0.032) | 0.133 |
| Plaque area, mm^2^ | 12M | 12.11 ± 1.040 | 11.83 ± 0.816 | 0.282  (-2.107 - 2.673) | 0.812 | 14.06 ± 1.122 | 13.27 ± 1.049 | 0.792  (-1.757 - 3.341) | 0.538 |
|  | 24M | 11.05 ± 0.789 | 10.32 ± 0.681 | 0.693  (-1.240 - 2.626) | 0.477 | 13.03 ± 0.837 | 11.17 ± 0.770 | 1.854  (-0.091 - 3.800) | 0.061 |
| Plaque gray scale median | 12M | 59.92 ± 7.504 | 64.46 ± 5.891 | -4.543  (-21.80 - 12.71) | 0.598 | 59.38 ± 5.772 | 58.89 ± 5.362 | 0.488  (-12.551 - 13.53) | 0.940 |
|  | 24M | 48.46 ± 3.527 | 50.47 ± 3.063 | -2.017  (-10.71 - 6.680) | 0.645 | 50.11 ± 3.443 | 53.20 ± 3.163 | -3.087  (-11.11 - 4.938) | 0.447 |

Values are adjusted for the baseline values using analysis of covariance.
