# Supplementary material for: The Effect of Sitagliptin on Carotid Artery Atherosclerosis in Type 2 Diabetes: The PROLOGUE Randomized Controlled Trial
Source: PLoS Med. 2016 Jun 28;13(6):e1002051. doi: 10.1371/journal.pmed.1002051 (PMC4924847; doi:10.1371/journal.pmed.1002051)
Supplement: S2 Table — (DOCX) [file pmed.1002051.s003.docx]

**S2 Table. Physiological and biochemical parameters at 12 months.**

|  | Sitagliptin  (n = 222)  Baseline-adjusted means ± SE | Conventional  (n = 220)  Baseline-adjusted means ± SE | Group Difference  Baseline-adjusted means (95%CI) | *p*-Value |
| --- | --- | --- | --- | --- |
| Body weight (kg) | 64.7 ± 0.22 | 64.6 ± 0.23 | 0.117 (-0.439 - 0.674) | 0.678 |
| Body mass index (kg/m^2^) | 25.0 ± 0.09 | 25.0 ± 0.09 | 0.045 (-0.174 - 0.264) | 0.689 |
| Waist circumstances (cm) | 89.9 ± 0.43 | 89.9 ± 0.43 | 0.005 (-1.071 - 1.081) | 0.993 |
| Systolic blood pressure (mmHg) | 128.9 ± 1.11 | 130.1 ± 1.12 | -1.081 (-3.872 - 1.710) | 0.447 |
| Diastolic blood pressure (mmHg) | 71.7± 0.78 | 73.2 ± 0.79 | -1.434 (-3.408 - 0.540) | 0.154 |
| Pulse rate (bpm) | 69.9 ± 0.76 | 71.4 ± 0.75 | -1.505 (-3.392 - 0.382) | 0.117 |
| HbA1c (%) | 6.56 ± 0.04 | 6.67 ± 0.04 | -0.105 (-0.209 - -0.001) | 0.047 |
| Fasting plasma glucose (mmol/L) | 7.24 ± 0.15 | 7.12 ± 0.14 | 0.122 (-0.236 - 0.480) | 0.502 |
| HOMA-β (%) | 78.5 ± 7.0 | 75.4 ± 6.7 | 3.084 (-13.92 - 20.09) | 0.721 |
| HOMA-R | 4.45 ± 0.75 | 4.52 ± 0.72 | -0.062 (-1.888 - 1.763) | 0.946 |
| Insulin (μU/mL) | 15.1 ± 1.90 | 13.3 ± 1.83 | 1.826 (-2.795 - 6.448) | 0.437 |
| 1,5-anhydroglucitol,1,4-anhydro(-D)-glucitol (μmol/L) | 105.4 ± 2.76 | 98.8 ± 2.75 | 6.558 (-0.266 - 13.38) | 0.059 |
| High molecular weight adiponectin (μmol/L) | 0.165 ± 0.01 | 0.196 ± 0.01 | -0.030 (-0.052 - -0.008) | 0.008 |
| Low-density lipoprotein cholesterol (mmol/L) | 2.46 ± 0.04 | 2.49 ± 0.05 | -0.031 (-0.141 - 0.079) | 0.577 |
| High-density lipoprotein cholesterol (mmol/L) | 1.33 ± 0.02 | 1.36 ± 0.02 | -0.030 (-0.070 - 0.009) | 0.127 |
| Triglyceride (mmol/L) | 1.50 ± 0.06 | 1.54 ± 0.06 | -0.038 (-0.177 - 0.101) | 0.590 |
| Small dense low density lipoprotein cholesterol (mmol/L) | 0.87 ± 0.03 | 0.91 ± 0.03 | -0.036 (-0.110 - 0.039) | 0.348 |
| Remnant-like particles cholesterol (mmol/L) | 0.13 ± 0.006 | 0.14 ± 0.006 | -0.014 (-0.030 - 0.002) | 0.086 |
| Malondialdehyde-modified low-density lipoprotein (U/L) | 118.1 ± 2.63 | 114.8 ± 2.60 | 3.312 (-3.187 - 9.812) | 0.316 |
| Aspartate aminotransferase (IU/L) | 25.6 ± 0.62 | 25.5 ± 0.63 | 0.079 (-1.489 - 1.648) | 0.921 |
| Alanine aminotransferase (IU/L) | 24.6 ± 0.81 | 24.3 ± 0.81 | 0.254 (-1.775 - 2.283) | 0.806 |
| Lactic dehydrogenase (IU/L) | 206.1 ± 2.3 | 205.8 ± 2.3 | 0.369 (-5.415 - 6.155) | 0.900 |
| Amylase (IU/L) | 84.1 ± 2.8 | 79.2 ± 2.9 | 4.847 (-2.591 - 12.28) | 0.199 |
| Blood urea nitrogen (mmol/L) | 6.13 ± 0.11 | 6.20 ± 0.11 | -0.064 (-0.336 - 0.207) | 0.641 |
| Serum creatinine (μmol/L) | 77.0 ± 0.73 | 75.5 ± 0.75 | 1.445 (-0.384 - 3.275) | 0.121 |
| Estimated glomerular filtration rate (mL/min/1.73m²) | 65.5 ± 0.66 | 66.7 ± 0.67 | -1.216 (-2.880 - 0.448) | 0.152 |
| Cystatin-C (μmol/L) | 0.079 ± 0.001 | 0.078 ± 0.001 | 0.001 (-0.002 - 0.046) | 0.551 |
| Urinary albumin/creatinine ratio (mg/g) | 81.1 ± 15.8 | 52.8 ± 16.0 | 28.36 (-11.1 - 67.8) | 0.158 |
| Uric acid (μmol/L) | 346.3 ± 4.46 | 347.2 ± 4.53 | -0.880 (-12.09 - 10.32) | 0.877 |
