# Supplementary material for: The Effect of Sitagliptin on Carotid Artery Atherosclerosis in Type 2 Diabetes: The PROLOGUE Randomized Controlled Trial
Source: PLoS Med. 2016 Jun 28;13(6):e1002051. doi: 10.1371/journal.pmed.1002051 (PMC4924847; doi:10.1371/journal.pmed.1002051)
Supplement: S3 Table — (DOCX) [file pmed.1002051.s004.docx]

**S3 Table.** **Doses of sitagliptin during the study, no. (%)**

| Dose (mg/day) | Baseline | 12 months | 24 months |
| --- | --- | --- | --- |
| 25 | 22 (10.3) | 13 (6.4) | 14 (7.3) |
| 50 | 190 (89.2) | 134 (65.7) | 116 (60.4) |
| 100 | 1 (0.5) | 57 (27.9) | 62 (32.3) |
| Mean ± SD | 48 ± 8 | 62 ± 24 | 64 ± 26 |
