# Supplementary material for: The Effect of Sitagliptin on Carotid Artery Atherosclerosis in Type 2 Diabetes: The PROLOGUE Randomized Controlled Trial
Source: PLoS Med. 2016 Jun 28;13(6):e1002051. doi: 10.1371/journal.pmed.1002051 (PMC4924847; doi:10.1371/journal.pmed.1002051)
Supplement: S1 Text — (DOCX) [file pmed.1002051.s005.docx]

**Clinical Study Protocol**

**“The Effect of a DPP-4 Inhibitor on Vascular Diseases”**

**PROLOGUE
Program of vascular evaluation under
glucose control by DPP-4 inhibitor**

**― Ver6.1 ―**

⭘ Chief researcher

Koichi Node

Professor, Department of Cardiovascular Medicine, Saga University Faculty of Medicine

Toyoaki Murohara

Professor, Department of Cardiology, Nagoya University Graduate School of Medicine

⭘ Study Group Secretariat

Jun-ichi Oyama

Department of Cardiovascular Medicine, Saga University Faculty of Medicine

5-1-1 Nabeshima, Saga, 849-8501, Japan

⭘ Planned period of the study

Date of approval of the ethics review committee until September 30, 2016

⭘ Date of preparation

Ver. 1.0 prepared on September 19, 2010

Ver. 2.0 prepared on April 11, 2011

Ver. 3.0 prepared on May 1, 2012

Ver. 4.0 prepared on June 19, 2012

Ver. 4.1 prepared on February 22, 2013

Ver. 5.0 prepared on April 1, 2014

Ver. 6.0 prepared on August 19, 2014

Ver. 6.1 prepared on January 27, 2015

**<Table of Contents>**

[1. Background of the Study 2](#_Toc429120520)

[2. Objectives 2](#_Toc429120521)

[3. Summary of the Study Drug (For details, see Appendix 1) Package insert of sitagliptin.) 2](#_Toc429120522)

[Sitagliptin phosphate hydrate 2](#_Toc429120523)

[4. Subjects 4](#_Toc429120524)

[5. Methods for Providing an Explanation to the Patient and Obtaining Consent 5](#_Toc429120525)

[6. Method of the Study 5](#_Toc429120526)

[7. Endpoints 8](#_Toc429120527)

[8. Observations and Tests 9](#_Toc429120528)

[9. Criteria for Discontinuation 11](#_Toc429120529)

[10. Handling at the Onset of AEs 12](#_Toc429120530)

[11. Reporting Protocol Deviations 13](#_Toc429120531)

[12. Completion, Discontinuation and Interruption of the Study 13](#_Toc429120532)

[13. Period of the Study 14](#_Toc429120533)

[14. Data Tabulations and Statistical Analysis Methods 14](#_Toc429120534)

[15. Target Sample Size and Rationale for Determination 15](#_Toc429120535)

[16. Consideration for the Human Rights and Safety/Disadvantages of the Patients 16](#_Toc429120536)

[17. Burden of Expenses for the Patients 17](#_Toc429120537)

[18. Compensation for Health Injuries and Participation in Insurance 17](#_Toc429120538)

[19. Handling of the Good Clinical Practice (GCP) and Declaration of Helsinki 17](#_Toc429120539)

[20. Retention of Records 17](#_Toc429120540)

[21. Audits 17](#_Toc429120541)

[22. Publication of Study Results 18](#_Toc429120542)

[23. Study Organization 18](#_Toc429120543)

[24. Study Funds and Conflict of Interest 20](#_Toc429120544)

[25. Revision of the Protocol, etc. 20](#_Toc429120545)

[26. List of References/ Literature 21](#_Toc429120546)

Appendix 1) Package insert of sitagliptin

Appendix 2) Event evaluation criteria

Appendix 3) Manual for procedures for carotid artery echography

Appendix 4) Manual for procedures for FMD

Appendix 5) Manual for procedures for echocardiography

**Clinical Study**

**“The Effect of a DPP-4 Inhibitor on Vascular Diseases”**

# 1. Background of the Study

Diabetes mellitus (DM) is an important risk factor for macroangiopathy. In patients with type 2 diabetes mellitus (T2DM), the incidence of coronary artery disease increases by 2- to 4-fold of that in patients without DM.

Dipeptidyl peptidase-4 (DPP-4) inhibitors are anti-hyperglycemic drugs with a plasma glucose-dependent insulinotropic action and glucagon secretion inhibitory action that inhibit DPP-4, an enzyme breaking down incretin hormones namely glucagon-like peptide-1 (GLP-1) and glucose-dependent insulinotropic peptide (GIP), thereby increasing active incretin levels. Since they exert their effects plasma glucose-dependently, DPP-4 inhibitors have been reported to cause few adverse drug reactions (ADRs) such as hypoglycemia.

# 2. Objectives

To compare the effect to prevent the progression of arteriosclerosis between the DPP-4 inhibitor group and conventional group without DPP-4 inhibitors as measured by carotid artery intima-media thickness (IMT) using carotid artery echography. Also, to examine the effects on cardiovascular function and blood biomarkers. For a DPP-4 inhibitor, sitagliptin will be used.

# 3. Summary of the Study Drug (For details, see Appendix 1) Package insert of sitagliptin.)

# Sitagliptin phosphate hydrate

## (1) Dosage form and dosage

Sitagliptin phosphate hydrate 25 mg, 50 mg and 100 mg tablets

## (2) Characteristics and mechanism of action

Sitagliptin inhibits DPP-4, an enzyme breaking down incretin, which acts to keep plasma glucose at constant levels to promote insulin secretion when plasma glucose is high and prevent glucagon production, thereby controlling plasma glucose.

## (3) Indication

T2DM

However, it is limited to the case where adequate response is not achieved by any of the following therapies:

(i) Diet/exercise therapy only;

(ii) Diet/exercise therapy plus the use of sulfonylurea;

(iii) Diet/exercise therapy plus the use of thiazolidines; or

(iv) Diet/exercise therapy plus the use of biguanides.

## (4) Dosage and administration

The usual adult dosage is 50 mg of sitagliptin administered orally once daily.

When the effect is inadequate, the dosage may be increased up to 100 mg once daily while monitoring the clinical course.

## (5) Contraindications: (Sitagliptin is contraindicated in the following patients.)

(i) Patients with a history of hypersensitivity to any of the ingredients of this drug

(ii) Patients with severe ketosis, diabetic coma, precoma, or type 1 DM

(iii) Patients with severe renal impairment including patients requiring hemodialysis or peritoneal dialysis

(iv) Patients with severe infection, in a perioperative condition or with serious trauma

## (6) ADRs

Clinical studies (clinical trials)

In a Japanese clinical study, ADRs occurred in 96 of 1,190 patients (8.1%). Common ADRs included hypoglycemia in 17 patients (1.4%) and constipation in 12 patients (1.0%). Treatment-related abnormal changes in laboratory values occurred in 49 of 1,188 patients (4.1%). Common abnormal changes included ALT (GPT) increased in 18 out of 1,188 patients (1.5%), AST (GOT) increased in 12 out of 1,188 patients (1.0%) and γ-GTP increased in 10 out of 1,188 patients (0.8%).

1) Clinically significant ADRs

(i) Anaphylactic reaction (incidence unknown)^Note)^: Anaphylactic reaction may occur. Therefore, the patient should be monitored, and if any abnormality is noted, treatment should be discontinued and appropriate therapeutic measures should be taken.

(ii) Oculomucocutaneous syndrome (Stevens-Johnson syndrome), dermatitis exfoliative (incidence unknown for both)^Note)^: Oculomucocutaneous syndrome (Stevens-Johnson syndrome) and dermatitis exfoliative may occur. Therefore, if these symptoms occurred, treatment should be discontinued, and appropriate therapeutic measures should be taken.

(iii) Hypoglycemia: Hypoglycemia (2.1%) occurred when sitagliptin was administered in combination with other antidiabetic drugs. Also, even when sitagliptin was not administered in combination with other antidiabetic drugs, hypoglycemia (1.0%) was reported. If symptoms of hypoglycemia are noted, appropriate therapeutic measures such as taking glucose-containing foods should be taken.

^Note)^ The events were reported in overseas countries.

2) Other ADRs

If the following symptoms or abnormalities occur, appropriate therapeutic measures such as discontinuing treatment should be taken:

| Type/Incidence | 0.1 to < 2% | Incidence unknown^Note)^ |
| --- | --- | --- |
| Nervous system disorders | Dizziness, hypoaesthesia |  |
| Eye disorders | Worsening of diabetic retinopathy |  |
| Cardiac disorders | Supraventricular extrasystoles |  |
| Respiratory, thoracic and mediastinal disorders |  | Upper respiratory tract infection, nasopharyngitis |
| Gastrointestinal disorders | Abdominal discomfort, bloating, abdominal pain, constipation, meteorism, gastritis, reflux oesophagitis, stomach discomfort, stomatitis | Pancreatitis |
| Skin and subcutaneous tissue disorders | Rash | Cutaneous vasculitis, urticaria, angioedema |
| General disorders | Hunger, oedema |  |
| Investigations | Electrocardiogram T wave amplitude decreased, weight increased, red blood cell count decreased, white blood cell count increased, ALT (GPT) increased, AST (GOT) increased, γ-GTP increased, CK (CPK) increased, blood cholesterol increased, blood uric acid increased, blood creatinine increased, low density lipoprotein increased, blood triglycerides increased, protein urine present |  |

^Note)^ The events were reported in overseas countries.

# 4. Subjects

## (1) Inclusion criteria

1) T2DM patients with poorly controlled plasma glucose (HbA1c ≥ 5.8% or < 9.0%: JDS, ≥ 6.2% or 9.4%: National Glycohemoglobin Standardization Program (NGSP)) despite diet/exercise therapy, or treatment with oral antidiabetic drugs other than DPP-4 inhibitors for ≥ 3 months in addition to diet/exercise therapy.

2) Outpatients aged 30 years or older (male and female)

3) Patients who provided written consent for participation in the study

## (2) Exclusion criteria

Patients who meet any of the following criteria will not be included in the study:

1) Patients with type 1 DM

2) Patients with a history of severe ketosis, diabetic coma or precoma within the past 6 months

3) Patients with severe infection, in a perioperative condition or with serious trauma

4) Patients with severe renal impairment (glomerular filtration rate: eGFR < 30 mL/min or dialysis patients)

5) Patients who developed myocardial infarction, angina pectoris or underwent percutaneous transluminal coronary angioplasty or bypass surgery, developed cerebral infarction, cerebral hemorrhage, subarachnoid hemorrhage or transient ischemic attack within 3 months prior to the start of the study

6) Patients with moderate or more severe heart failure (NYHA functional class III or IV)

7) Patients who are on DPP-4 inhibitors or GLP-1 analogues at the time of study initiation

8) Patients who are on insulin at the time of study initiation

9) Patients who are pregnant, lactating, of childbearing potential, or planning to become pregnant

10) Patients with a history of hypersensitivity to the study drug

11) Patients who are determined by the investigator to be not suitable for other reasons such as malignancies

# 5. Methods for Providing an Explanation to the Patient and Obtaining Consent

(1) After giving the informed consent document approved by the institutional review board (IRB) or ethics committee at each site and an adequate explanation using the informed consent document to the patient, written voluntary consent of the patient for participation in the study should be obtained.

(2) When information on efficacy, safety and other relevant matters is obtained that may affect the patient’s consent or when changes in the protocol and other relevant items are made that may affect the patient’s consent, the information should be promptly provided to the patient, his/her intention as to whether or not to continue participation in the study should be verified beforehand, prior approval of the IRB or ethics committee at each site should be obtained, the informed consent document, etc., should be then revised, and the patient’s consent should be received again.

(3) A patient may voluntarily withdraw his/her consent to participate in the study at any time during study enrollment. The investigator should check the level of the patient’s withdrawal as to whether it is withdrawal from participation in the overall study (Data from the applicable patient may not be used) or deviation from the treatment method in the study (Part or all of data from the applicable patient may be used).

# 6. Method of Study

## (1) Study type/design

Phase: Clinical study using a marketed drug

Design: Prospective, randomized, open-label and blinded-endpoint evaluation (PROBE)

Category: Investigator-initiated clinical study

## (2) Outline of the study

| **Target plasma glucose control level: HbA1c (JDS) < 5.8% or fasting plasma glucose < 110 mg/dL** | | | | |  |
| --- | --- | --- | --- | --- | --- |
|  | | | | | |
| **Group A: Sitagliptin group** | | | | | |
|  | | | | Not achieving the target: Increase the dose of the concomitant medication, etc. | |
|  | | | Not achieving the target: Increase the dose of sitagliptin to 100 mg | | |
|  | | Sitagliptin 50 mg^*1,*2^ | | | |
| 5.8% ≤ HbA1c (JDS) < 9.0%   1. Diet/exercise therapy only 2. Diet/exercise therapy + oral antidiabetic drugs (receiving treatment with antidiabetic drugs excluding DPP-4 inhibitors) | ^*1^ When patients who are on glinide are assigned to Group A, glinide is discontinued and switched to sitagliptin. The doses of the antidiabetic drugs used may be reduced at the start of the study.  ^*2^ The specific dosage regimen is described in Section 6. Method of the Study, (4) Treatment schedule. | | | | |
| **Group B: Conventional group** | | Oral antidiabetic drugs excluding DPP-4 inhibitors^*2^ | | | |

**0M 3M 6M 12M 24M**

## (3) Planned duration of study enrollment by patients

Two years from the enrollment in the study

## (4) Treatment schedule

### 1) Before the start of the study

After selecting the subjects from past medical records, the investigator will give an explanation of the contents of the study to the subjects using the informed consent document and receive written consent from them.

The subjects will be assigned to either the non-drug therapy or drug therapy group according to the treatment method at the time of enrollment. Using the background characteristics of the subjects in each group (statin use, age, gender, blood pressure, and baseline HbA1c and IMT levels) as prognostic predictors, computerized randomization will be performed using a minimization method so that the prognosis predictors will be equally assigned to Group A: sitagliptin group and Group B: conventional group.

### 2) Duration of the study: 2 years

The investigator will start treatment with sitagliptin in Group A and continue conventional treatment excluding DPP-4 inhibitors in Group B in accordance with randomization results.

Group A: Treatment in the sitagliptin group will be carried out using the following procedures:

(i) Treatment is started with sitagliptin 50 mg

(ii) In patients who are on glinide prior to randomization, glinide is discontinued and switched to sitagliptin. The dosage of antidiabetic drugs that are used may be reduced at the start of the study.

(iii) If HbA1c levels are ≥ 5.8% at Month 3, the dose of sitagliptin is increased to 100 mg.

(iv) If HbA1c (JDS) levels are ≥ 5.8% at 3 months thereafter, treatment with a concomitant medication (excluding glinide) is added in patients who are not on drug therapy at enrollment, and the dosage of a concomitant medication will be increased in patients who are on drug therapy. The treatment will be continued until the therapeutic goal of HbA1c levels < 5.8% is achieved.

^Note 1)^ For subjects with moderate renal impairment (eGFR ≥ 30 to < 50 mL/min), the treatment may be started with a dose reduction from sitagliptin 25 mg at the discretion of the primary physician. If the therapeutic goal is not achieved, the dose may be increased up to 50 mg upon confirming tolerability.

^Note 2)^ When sitagliptin is administered in combination with sulfonylurea (SU), attention should be paid to the fact that the following recommendation was presented by the Japan Diabetes Society and Japan Association for Diabetes Education and Care in April 2010:

<Recommendation>

The doses should be reduced to ≤ 2 mg/day for patients who are on Amaryl^®^ (glimepiride) > 2 mg/day, ≤ 1.25 mg/day for patients who are on Euglucon^®^ (Daonil^®^) > 1.25 mg/day, and ≤ 40 mg/day for patients who are on Glimicron^®^ > 40 mg/day. If plasma glucose control is inadequate after treatment in combination with sitagliptin, the dose of SU should be, as necessary, increased, and if hypoglycemia occurs, the SU dose should be decreased further. When patients are treated with SU at doses lower than the abovementioned ones in whom plasma glucose is poorly controlled, treatment should be continued in combination with sitagliptin, and the SU doses should be reduced as necessary if plasma glucose improves.

Group B: Treatment in the conventional group will be carried out using the following procedures:

In patients who are not on drug therapy at enrollment, treatment will be started with a drug excluding DPP-4 inhibitors. In patients who are on drug therapy, more vigorous treatment, such as increasing the dose of the drug that is being used or switching to another drug excluding DPP-4 inhibitors, will be instituted. The treatment will be continued until the therapeutic goal of HbA1c (JDS) levels < 5.8% is achieved.

## (5) Dosage form/content, description, package, labeling and storage method of the study drug

See **Appendix 1) Package insert of sitagliptin.**

## (6) Rules for concomitant medications (therapies)

### 1) Prohibited concomitant medications

Group A: DPP-4 inhibitors except sitagliptin, GLP-1 analogues, glinide and insulin

Group B: DPP-4 inhibitors, GLP-1 analogues and insulin

### 2) Restricted concomitant medications (therapies)

No new start, discontinuation or dose reduction shall be implemented for diet and exercise therapy, food for specified health use, etc. during the study period. There is no restriction for drugs for the treatment of complications (e.g., antiplatelet drugs, hypotensive drugs and antihyperlipidemic drugs), but no dosage change, discontinuation, or new addition of drugs should be made to the extent possible during the treatment period.

# 7. Endpoints

## (1) Primary endpoint

Percent change in mean common carotid artery (CCA)-IMT at Month 24 from baseline as measured by carotid artery echography

## (2) Secondary endpoints

### 1) Observed IMT values at baseline, Month 12 and Month 24 as measured by carotid artery echography (CCA, carotid bulbs and internal carotid artery [ICA]) and the change/percent change from baseline (excluding the percent change in mean CCA-IMT at Month 24 from baseline)

Mean IMT, maximum IMT, plaque area, and grey scale value

### 2) Observed values at baseline, Month 12 and Month 24 and changes/percent changes from baseline for the following laboratory tests:

NT-proBNP, high-sensitive CRP, 1,5AG, small particle low-density lipoprotein (LDL), high molecular weight adiponectin, RLP-C and MDA-LDL

### 3) Observed values at baseline, Months 3, 6, 12 and 24, and changes/percent changes from baseline for the following laboratory tests:

HbA1c, fasting plasma glucose, insulin, serum lipid (TC, HDL-C and TG), serum creatinine, urinary albumin excretion (corrected for creatinine execretion rate), eGFR (converted value), HOMA-β (converted value), HOMA-R (converted value), and cystatin C.

### 4) Observed values at baseline, Month 12 and Month 24 and changes/percent changes from baseline for the following cardiovascular function tests:

Echocardiography (systolic and diastolic function, left atrial dimension, and left ventricular mass index), endothelium-dependent vasodilator response (FMD), pulse wave velocity (PWV), cardio ankle vascular index (CAVI) and atherosclerosis index (AI)

### 5) Observed values at baseline, Months 3, 6, 12 and 24, and changes/percent changes from baseline for the following items, and the incidence of significant clinical symptoms:

Blood pressure (outpatient), body weight and incidence of significant changes in body weight, and incidence of hypoglycemia symptoms (when moderate or more severe hypoglycemia symptoms as defined below are noted)

<Definition of hypoglycemia symptoms>

Mild: Almost or fully active to be able to carry out daily and physical activities, and symptoms not requiring treatment

Moderate: Restricted in daily and physical activities, but symptoms not requiring treatment or care by family, etc.

Severe: Symptoms requiring treatment or care by family, etc.

Very severe: Symptoms requiring medical treatment (e.g., requiring to call an ambulance or treatment at the hospital)

### 6) The following items during the treatment period (24 months) from baseline:

Composite endpoint (cardiovascular death, non-fatal myocardial infarction and stroke, renal event and insulin initiation) and each endpoint

* For the evaluation methods for events, see **Appendix 2) Event evaluation criteria**.

# 8. Observations and Tests

## (1) Patient background: The following items will be investigated at baseline:

Identification (ID) number, gender, birth date, body height, body weight, abdominal circumference, duration of DM, complications (presence or absence of renal disease, liver disease, cerebro- and cardiovascular diseases, hypertension and hyperlipidemia), past history (presence or absence of current or past history of cardiovascular diseases), drugs used (antidiabetic drugs, hypotensive drugs, antihyperlipidemic drugs and antithrombotic drugs), presence or absence of alcohol drinking/smoking and study treatment compliance

## (2) Outpatient casual blood pressure/pulse rate: Baseline and Months 3, 6, 12 and 24

Blood pressure and pulse rate will be measured with patients in sitting position after resting for at least 5 minutes, as a rule. The time of measurements should be at certain time points throughout the treatment period to the extent possible.

## (3) Echocardiography/chest radiography: Baseline and Months 12 and 24

## (4) Carotid artery echography (IMT): Baseline and Months 12 and 24

For details, see **Appendix 3) Manual for procedures for carotid artery echography.**

## (5) Cardiovascular function test

Echocardiography, FMD, PWV, CAVI and AI

(Optional items: Measured at feasible sites at baseline and Months 12 and 24)

For the details of FMD, see **Appendix 4) Manual for procedures for FMD**.

For the details of echocardiography, see **Appendix 5) Manual for procedures for echocardiography**.

## (6) Hematology: Baseline and Months 3*, 6*, 12 and 24 (*Moths 3 and 6 are optional)

Red blood cell, white blood cell, hemoglobin, hematocrit and platelet

## (7) Blood biochemistry: Baseline and Months 3*, 6*, 12 and 24 (*Moths 3 and 6 are optional except for HbA1c)

AST, ALT, LDH, serum creatinine, BUN, uric acid, Na, K, Cl, TC, HDL-C, TG, plasma glucose, HbA1c, insulin, cystatin C (at the time of decreased renal function) and amylase

## (8) Special blood biochemistry * Centralized measurement using study expenses: Baseline and Months 12 and 24

NT-proBNP, high-sensitive CRP, 1,5AG, small particle LDL, high molecular weight adiponectin, RLP-C, and MDA-LDL

## (9) Urinalysis: Baseline and Months 3*, 6*, 12 and 24 (*Moths 3 and 6 are optional)

Qualitative (urinary sugar, urine protein and urinary occult blood) and urinary albumin excretion (corrected by creatinine)

## (10) Event investigation

Date of onset, outcome and date of outcome, category of event, and findings and comments by the investigator

* For the evaluation methods for events, see **Appendix 2) Event evaluation criteria**.

## (11) Verification of adverse events (AEs) (excluding the events)

Adverse events (AEs) include ADRs as well as abnormal laboratory values. An AE term, date of onset, seriousness, therapeutic action, outcome, date of outcome, causal relationship with the study drug, and concomitant medications likely to be suspected drugs should be recorded in medical records and a case report form (CRF). A follow-up investigation should be carried out if needed.

<Severity of AE>

1) Mild: The treatment can be continued without treatment.

2) Moderate: The treatment can be continued with some treatment.

3) Severe: The treatment is or should be discontinued.

Seriousness assessment is defined in the Section **10.-(2) Reporting serious adverse events (SAEs)**, and if applicable, SAEs should be promptly reported.

## (12) Test schedule

⭘△, Implement before study drug administration; ⚫▲, Implement after study drug administration; and △▲, Optional items.

^Note)^ In principle, blood should be collected in the fasting state. The range of a visit is within ± one month.

|  | Baseline | Study period | | | | |
| --- | --- | --- | --- | --- | --- | --- |
| Time point |  | 0M | 3M | 6M | 12M | 24M |
| Informed consent | ⭘ |  |  |  |  |  |
| Patient background | ⭘ |  |  |  |  |  |
| Patient enrollment, randomization^*1^ | ⭘ |  |  |  |  |  |
| Body weight |  | ⭘ | ⚫ | ⚫ | ⚫ | ⚫ |
| Abdominal circumference |  | ⭘ |  |  | ⚫ | ⚫ |
| Treatment compliance |  | ⭘ | ⚫ | ⚫ | ⚫ | ⚫ |
| Outpatient casual blood pressure/pulse rate |  | ⭘ | ⚫ | ⚫ | ⚫ | ⚫ |
| HbA1c |  | ⭘ | ⚫ | ⚫ | ⚫ | ⚫ |
| Hematology/urinalysis^*3^ |  | ⭘ | ▲ | ▲ | ⚫ | ⚫ |
| Special biochemistry^*4^ |  | ⭘ |  |  | ⚫ | ⚫ |
| Echocardiography/chest radiography |  | ⭘ |  |  | ⚫ | ⚫ |
| IMT |  | ⭘^*2^ |  |  | ⚫ | ⚫ |
| Cardiovascular function test^*5^ |  | △ |  |  | ▲ | ▲ |
| Event/AE investigation |  | ⭘ | ⚫ | ⚫ | ⚫ | ⚫ |

^*1^ Randomization factors: Therapy (non-drug therapy or drug therapy) before the start of the study, gender, age, statin use, blood pressure, HbA1c (JDS), cIMT (higher far wall CCA-IMT value between the right and left CCA)

^*2^ IMT at 0M may be substituted with IMT at randomization

^*3^ Hematology: Red blood cell, white blood cell, hemoglobin, hematocrit, platelet, AST, ALT, LDH, serum creatinine, BUN, uric acid, Na, K, Cl, TC, HDL-C, TG, plasma glucose, insulin, cystatin C (at the time of decreased renal function) and amylase

Urinalysis: Qualitative (urinary sugar, urine protein and urinary occult blood) and urinary albumin excretion (corrected by creatinine)

^*4^ Special blood biochemistry: NT-proBNP, high-sensitive CRP, 1,5AG, small particle LDL, high molecular weight adiponectin, RLP-C, and MDA-LDL

^*5^ Cardiovascular function test (echocardiography, FMD, PWV, CAVI and AI)

# 9. Criteria for Discontinuation

When it is determined that the study cannot be continued because of the belowmentioned reasons, the investigator should discontinue study treatment, record the date/period of withdrawal/dropout, reason for withdrawal/dropout and clinical course in the medical records and CRF, and perform tests required at the time of withdrawal/dropout to evaluate efficacy and safety:

(1) When moderate or more severe hypoglycemia is noted after treatment initiation;

(2) When plasma glucose control has markedly worsened (HbA1c ≥ 10% in two consecutive measurements) after treatment initiation;

(3) When the patient has requested to withdraw from the study participation or their consent;

(4) When the patient is found to be ineligible after enrollment;

(5) When continuation of the study treatment is determined to be undesirable due to progression of the primary disease or complications;

(6) When continuation of the study is difficult due to AEs;

(7) When pregnancy is identified;

(8) When treatment compliance is markedly poor (when it is judged that < 75% or > 120% of the planned total number of doses of the study drug is taken); and

(9) When the investigator has determined that it is appropriate to discontinue the study due to other reasons.

# 10. Handling at the Onset of AEs

## (1) Action for the patients at the onset of AEs

When an AE is reported, the investigator should immediately take appropriate therapeutic action and record it in the medical records and CRF without inconsistency. When the study treatment is discontinued or treatment is required for the AE, such a fact should be notified to the patient.

## (2) Reporting serious adverse events (SAEs)

1) Definition of serious adverse events (SAEs)

(i) An event that results in death;

(ii) An event that is life-threatening;

(iii) An event that requires inpatient hospitalization or prolongation of existing hospitalization;

(iv) An event that results in persistent or significant disability/incapacity; or

(v) An event that is a congenital anomaly/birth defect.

2) AEs subject to reporting

All SAEs during the study period and SAEs suspected to be related to the study drug after the end (discontinuation) of the study should be reported.

3) When the onset of SAEs is identified, the investigator should promptly report it to the head of the study site and study representative.

4) The reporting periods will be, in principle, within 7 and 15 days according to the categories of seriousness and unknown/known in accordance with Article 273 of the Enforcement Regulations of the Pharmaceutical Affairs Law.

5) For marketed drugs, etc., reports should be made to the Ministry of Health, Labour and Welfare (MHLW) through the Pharmaceuticals and Medical Devices Safety Information Reporting System by the MHLW.

## (3) Other AEs

Other AEs should be recorded in CRFs using procedures described in the Section **8. Observations and Tests-(11) Verification of adverse events (AEs) and ADRs.**

# 11. Reporting Protocol Deviations

(1) The investigator shall not make any protocol deviation or change before obtaining prior consent of the study representative.

(2) The investigator may make protocol deviations or changes before obtaining prior consent of the study representative because of inevitable reasons such as emergency avoidance. At that time, the investigator should promptly submit the details and reason for the deviation or change and if a revision of the protocol and other relevant documents is needed, its proposal to the study representative, and obtain approval.

(3) In case deviation from the protocol occurs, the investigator shall record all deviations and their reasons and report them to the head of the participating site, to which he/she belongs, and the study representative using the form specified by the study site. The investigator shall retain their copies.

# 12. Completion, Discontinuation and Interruption of the Study

## (1) Completion of the study

At completion of the study, such a fact should be notified to the study representative.

## (2) Discontinuation or interruption of the study

1) When meeting the following items, the investigator should examine whether or not the study can be continued:

(i) When important information on the quality, safety or efficacy of the study drug is obtained;

(ii) When it is difficult to recruit patients and it is judged that the planned sample size cannot be reached;

(iii) When the objectives of the study are achieved (by an interim analysis, etc.) before the planned sample size or period is achieved; or

(iv) When the study representative has instructed a protocol amendment, etc. and such amendment is judged to be unacceptable.

2) When the study representative has advised or instructed study discontinuation, the study should be discontinued.

3) When discontinuation or interruption of the study is decided, such a fact and its reason should be promptly reported in writing to the head of the study site.

# 13. Period of the Study

Date of approval of the ethics review committee to September 30, 2016

Treatment observation period: December 31, 2014 (closing date of enrollment: September 30, 2012)

Study period: September 30, 2016 (publication of analysis and study results)

# 14. Data Tabulations and Statistical Analysis Methods

## (1) Data collection/tabulation

1) Procedures for data collection

The investigator should input case reports through WEB or send CRFs to the study secretariat. The study group secretariat should enter all data from each site and save them in an electronic medium.

2) Procedures for data accumulation

Prior to a data analysis, the study group secretariat should check the presence or absence of protocol deviations, their severity, dropping out from the study and its date, the patient’s willingness for using analysis data and other relevant matters for each patient, and then determine the following three analysis sets:

(i) Full analysis set (FAS)
Patients who were enrolled in this study and assigned to study treatment will be included in the full analysis set (FAS). However, patients with major protocol violation (e.g., consent not received and enrollment out of the contract period) will be excluded.

(ii) Per protocol set (PPS)
Patients in the FAS excluding those with the following major violation of protocol rules such as study treatment and concomitant therapies:

Violation of the inclusion criteria

Violation of the exclusion criteria

Violation of the use of the prohibited concomitant medications

Violation of the use of the prohibited concomitant therapies

(iii) Safety analysis set
Patients who were enrolled in this study, started treatment as assigned, and received part or all of the study treatment.

## (2) Analysis methods

### 1) Basic analysis policy

In all efficacy evaluations, an analysis using the full analysis set FAS will be the primary analysis, and as a reference, an analysis using the per protocol set (PPS) will be performed. Safety analyses will be carried out using the safety analysis set. The details are separately described in the statistical analysis plan.

### 2) Analysis of patient background

The distribution and summary statistics of patient background data in each analysis set will be calculated for each assigned group. For nominal variables and ordinal variables, the frequencies and proportions of categorical data will be presented for each assigned group. For continuous variables, summary statistics will be calculated for each assigned group. Tests to be used will be the chi-square-test (as necessary, Fisher’s exact test) for nominal variables, Wilcoxon rank-sum test for ordinal variables, and t-test for continuous variables (two-sided significance level of 0.05).

### 3) Analysis of the primary endpoint (IMT)

The primary objective of this study is to evaluate whether or not the percent change in IMT at Month 24, the primary endpoint significantly decreases in the sitagliptin group compared with that in the conventional group. A null hypothesis that the percent change in IMT between the two groups is equivalent in the primary analysis will be performed using analysis of covariance (ANCOVA). At that time, covariates will be randomization factors (statin use, age, gender, blood pressure, and baseline HbA1c and IMT). In addition, as sensitivity analysis, the time-course of IMT in each group will be presented, and longitudinal data will be analyzed using a linear mixed effects model to confirm that the results are similar to those of the ANCOVA. The significance level will be 0.05 for a two-sided test, and two-sided 95% confidence intervals will be calculated.

### 4) Analyses of the secondary endpoints

Analyses of the secondary endpoints will be performed for examining to complement the results of the primary analysis in the study. No multiplicity adjustment will be made in the analyses of the secondary endpoints. As necessary, comparisons between the groups will be carried out. The details are separately described in the statistical analysis plan.

### 5) Exploratory analysis

In addition to the above analyses, exploratory analyses such as a correlation analysis of each factor change, cause analysis for the onset of events, survival analysis, and multiple linear regression analysis will be conducted.

### 6) Interim analysis

Considering the current situation that continuing treatment for a long time without the use of DPP-4 inhibitors is becoming difficult and the vascular protection effects of DPP-4 inhibitors are starting to be reported, it is necessary to evaluate the results of this study as soon as possible from the viewpoint of welfare of patients. Consequently, an interim analysis will be performed one year after the end of patient enrollment. The details are separately described in the interim analysis plan.

# 15. Target Sample Size and Rationale for Determination

## (1) Target sample size

500 patients (250 patients in each group)

## (2) Rationale for determination

In CHICAGO study^1)^, in which the effect of thiazolidines and SU on CCA-IMT was investigated in T2DM patients, there was no significant difference in HbA1c up to Week 48 between the two groups, but the mean change in IMT was -0.001 mm in the pioglitazone group and +0.012 mm in the glimepiride group; thus, it was reported that the progression of CCA-IMT was significantly inhibited in the pioglitazone group.

At present, no study of the effect of DPP-4 inhibitors on CCA-IMT has been reported. However, assuming that sitagliptin has an effect of inhibiting the progression of CCA thickness equivalent to that of pioglitazone, and the change in CCA-IMT at Week 48 would be -0.001 ± 0.05 mm in the sitagliptin group and +0.012 ± 0.05 mm in the conventional group, the minimum number of patients necessary for detecting a significant difference between the two groups under conditions, i.e., a hazard ratio of 5% and power of 80%, is 464 patients in total or 232 patients in each group.

In addition, when the change in CHICAGO study is converted to a percent change, and the percent change in CCA-IMT is assumed to be -0.130 ± 6.5% in the sitagliptin group and 1.540 ± 6.5% in the conventional group, the minimum number of patients necessary for detecting a significant difference between the two groups under conditions, i.e., a hazard ratio of 5% and power of 80%, is 476 patients in total or 238 patients in each group.

Assuming a dropout of approximately 5%, the target number of patients to be enrolled is 500 patients in total or 250 patients in each group.

# 16. Consideration for the Human Rights and Safety/Disadvantages of the Patients

## (1) Consideration for human rights (protection of privacy)

1) When handling raw data and informed consent forms, etc. related to the conduct of the study, careful consideration should be paid to the protection of the confidentiality of the patients. In CRFs and other relevant documents to be submitted to the study group secretariat, ID numbers should be used. When study results are published, information that enables identification of patients will not be included. In response to the results of this study, samples may be used for further exploratory analyses, but data from the patients should not be used for purposes other than this.

2) When measurements, etc. are performed by sending samples from the patients to external parties, they will be anonymized such as using ID numbers to prevent identification of patients.

## (2) Consideration for safety/disadvantages

When AEs occur, appropriate treatment and therapeutic actions should be promptly implemented, and they should be reported in accordance with Section **10. Handling at the Onset of AEs**.

# 17. Burden of Expenses for the Patients

In this study, all tests excluding the special blood test and the study treatment will be carried out within the scope of usual health insurance treatment. The special blood test will be performed using the study expenses. Therefore, participation in this study will not increase a financial burden to the patients.

# 18. Compensation for Health Injuries and Participation in Insurance

## (1) Compensation for health injuries

Because this study will be conducted within the scope of authorization for approved drugs, no special compensation will be provided in the event of injury attributable to the study drug or other relevant matters. Thus, injuries will be handled the same as those and medical accidents occurred in usual medical care. Their compensation will be provided from the Drug Relief, R&D Promotion and Product Review by the Pharmaceuticals and Medical Devices Agency or products completed liability insurance.

## (2) Participation in insurance

Because this study will be conducted within the scope of authorization for approved drugs, in the event of injury attributable to the study drug or other relevant matters, compensation will be provided from the Drug Relief, R&D Promotion and Product Review by the Pharmaceuticals and Medical Devices Agency or products completed liability insurance. However, since the study design is an interventional study requiring randomization, the chief researchers are to be covered by the “liability insurance for clinical studies” handled by an insurance company on behalf of the investigators and subinvestigators.

# 19. Handling of the Good Clinical Practice (GCP) and Declaration of Helsinki

This study will comply with the ethical guidelines for clinical studies. Also, it will be implemented in compliance with the Declaration of Helsinki (revised in 2000).

# 20. Retention of Records

The representative study doctors should retain the essential documents related to the study and other relevant matters (copy of application documents, copies of applications/reports, enrollment numbers, informed consent forms, copies of CRFs, etc. other documents or records necessary for ensuring data reliability, etc.) up to 5 years after the publication of the study.

# 21. Audits

Audits are intended to determine whether or not this study is properly conducted and ensure the reliability of the data. The study organization will contract out audits of the study to an external party. When performing audits, records revealing the identities of the patients and secretes of confidential medical information should be kept to protect privacy. The investigators and study sites should provide records on the study for direct access upon request and collaborate in this at the time of audits and investigations by the ethics review committee.

Audit manager: Shinichiro Ueda, Director, Clinical Research Support Center, University of the Ryukyus Hospital

# 22. Publication of Study Results

## (1) Timing of publication

Around 2014

## (2) Methods of publication

Presentation at academic conferences and medical papers

# 23. Study Organization

## (1) Chief researcher

Koichi Node, Professor, Department of Cardiovascular Medicine, Saga University Faculty of Medicine

Toyoaki Murohara, Professor, Department of Cardiology, Nagoya University Graduate School of Medicine

## (2) Steering committee

| Cardiovascular | Hokkaido University | Department of Cardiovascular Medicine | Professor | Hiroyuki Tsutsui |
| --- | --- | --- | --- | --- |
| Cardiovascular | Akita University | Department of Cardiovascular and Respiratory Medicine | Professor | Hiroshi Ito |
| Cardiovascular | Dokkyo Medical University | Department of Cardiovascular Medicine | Professor | Teruo Inoue |
| Cardiovascular | Showa University | Department of Diabetes, Metabolism, and Endocrinology | Professor | Tsutomu Hirano |
| Cardiovascular | Juntendo University | Department of Cardiovascular Medicine | Professor | Hiroyuki Daida |
| Cardiovascular | Mie University | Department of Cardiology and Nephrology | Professor | Masaaki Ito |
| Cardiovascular | National Cerebral and Cardiovascular Center | Department of Clinical Medicine and Development | Director | Masafumi Kitakaze |
| Cardiovascular | Tokushima University | Department of Cardiovascular Medicine | Professor | Masataka Sata |
| Cardiovascular | Nagasaki University | Department of Cardiovascular Medicine | Professor | Koji Maemura |
| Diabetes | Tokushima University | Diabetes Therapeutics and Research Center | Professor | Munehide Matsuhisa |
| Diabetes | Kawasaki Medical School | Department of Internal Medicine | Professor | Kohei Kaku |
| Metabolism | Yokohama City University | Department of Endocrinology and Metabolism | Professor | Yasuo Terauchi |
| Neurology | Tokyo Women’s Medical University | Department of Medicine, Kidney Center | Professor | Kosaku Nitta |
| Neurology | Nippon Medical School | Department of Nephrology | Professor | Shuichi Tsuruoka |

## (3) Independent Efficacy and Safety Evaluation Committee

| Chairperson/Neurology | Kawasaki Medical School | Department of Nephrology and Hypertension | Professor | Naoki Kashihara |
| --- | --- | --- | --- | --- |
| Cardiovascular | Hyogo College of Medicine | Division of Cardiovascular Medicine and Coronary Heart Disease | Professor | Masaharu Ishihara |
| Diabetes | Tokyo Medical University | Department of Diabetes, Endocrinology, Metabolism and Rheumatology | Professor | Masato Odawara |
| Diabetes | University of the Ryukyus | Second Department of Medicine, Division of Endocrinology, Diabetes and Metabolism, Hematology, Rheumatology | Professor | Hiroaki Masuzaki |
| Stroke | Tokyo Women’s Medical University | Department of Neurology | Professor | Kazuo Kitagawa |
| Gastroenterology | Osaka University | Department of Gastroenterology | Associate Professor | Masahiko Tsujii |

## (4) Independent Data Monitoring Board

| Chairperson | Tokyo Medical University | Department of Cardiology | Professor | Akira Yamashina |
| --- | --- | --- | --- | --- |
|  | Jikei University School of Medicine | Division of Molecular Epidemiology | Professor | Mitsuyoshi Urashima |
|  | Dokkyo Medical University | Department of Cardiovascular Medicine, Nikko Medical Center | Professor | Takanori Yasu |

## (5) Working Group

| FMD (representative) | Hiroshima University | Research Institute for Radiation Biology and Medicine | Professor | Yukihito Higashi |
| --- | --- | --- | --- | --- |
| FMD | Shimane University | Department of General Medicine | Professor | Yutaka Ishibashi |
| FMD | Osaka City University | Department of Internal Medicine and Cardiology | Associate Professor | Kenei Shimada |
| PWV/CAVI/AI | Tokyo Medical University | Department of Cardiology | Professor | Hirofumi Tomiyama |
| IMT | University of Tsukuba | Department of Clinical Laboratory Medicine, Faculty of Medicine | Instructor | Tomoko Ishizu |
| Echocardiography | Tokushima University | Department of Cardiovascular Medicine/Ultrasound Examination Center | Instructor | Hirotsugu Yamada |

## (6) Measurement laboratories

Centralized measurement laboratories for IMT

Department of Clinical Laboratory Medicine, Faculty of Medicine, University of Tsukuba

Measurement laboratories for small particle LDL

Department of Diabetes, Metabolism, and Endocrinology, Internal Medicine Center, Showa University School of Medicine

## (7) Statistical Analysis Committee

Yasunori Sato, Instructor, Department of Global Clinical Research, Graduate School of Medicine, Chiba University

## (8) Joint study sites

Measurement of high molecular weight adiponectin

Fujirebio Inc. FR Building, 2-62-5 Nihonbashihamacho, Chuo-ku, Tokyo 103-0007

Measurement of NT-proBNP

Roche Diagnostics K.K. 2-6-1 Shiba, Minato-ku, Tokyo 105-0014

## (9) Study secretariat

Jun-ichi Oyama

Department of Cardiovascular Medicine, Saga University Faculty of Medicine

5-1-1 Nabeshima, Saga, 849-8501

<<Substitute secretariat>>

Hisako Fujimoto

Nouvelle Place Inc.

Yushima 1-chome Building, 1-6-3 Yushima, Bunkyo-ku, Tokyo 113-0034

Tel: 03-3814-1616  Fax: 03-3814-3388

E-Mail: prologue@n-place.co.jp

# 24. Study Funds and Conflict of Interest

This study will be conducted with a research grant from the Clinical Research Promotion Foundation. In the planning/implementation/reporting of this clinical study, there is no “possible conflict of interest” that may affect the results of the study or the interpretation of the results. The conduct of the study will not impair the rights or benefits of the patients.

# 25. Revision of the Protocol, etc.

When modifying (revising) the protocol or informed consent document, prior approval of the ethics committee is required.

# 26. List of References/ Literature

^1)^ Mazzone T *et al*; Effect of Pioglitazone Compared With Glimepiride on Carotid Intima-Media Thickness in Type 2 Diabetes. *JAMA* 2006: 296, 2572-2581
