# Supplementary material for: The Effect of Sitagliptin on Carotid Artery Atherosclerosis in Type 2 Diabetes: The PROLOGUE Randomized Controlled Trial
Source: PLoS Med. 2016 Jun 28;13(6):e1002051. doi: 10.1371/journal.pmed.1002051 (PMC4924847; doi:10.1371/journal.pmed.1002051)
Supplement: S2 Text — (DOC) [file pmed.1002051.s006.doc]

**SUMMARY OF PROTOCOL AMENDMENTS**

The initial version of the Protocol was written in Japanese and dated September 19, 2010. The current version of the Protocol (Ver 6.1) is dated January 27, 2015. It is only translated in English and ready for submission for the journal in English. Amendments are summarized below and highlighted.

***Ver. 1.0 documented on September 19, 2010***

***Ver. 2.0 documented on April 11, 2011***

*1. Exclusion criteria: “a patient using GLP-1 analogue”, “a patient with malignant tumor”*

*added.*

Previous Text

4. Subjects

(2) Exclusion criteria

Patients who meet any of the following criteria will not be included in the study:

1) Patients with type 1 DM

2) Patients with a history of severe ketosis, diabetic coma or precoma within the past 6 months

3) Patients with severe infection, in a perioperative condition or with serious trauma

4) Patients with severe renal impairment (glomerular filtration rate: eGFR < 30 mL/min or dialysis patients)

5) Patients who developed myocardial infarction, angina pectoris or underwent percutaneous transluminal coronary angioplasty and bypass surgery, developed cerebral infarction, cerebral hemorrhage, subarachnoid hemorrhage or transient ischemic attack within 3 months prior to the start of the study

6) Patients with moderate or more severe heart failure (NYHA functional class III or IV)

7) Patients who are on DPP-4 inhibitors at the time of study initiation

8) Patients who are on insulin at the time of study initiation

9) Patients who are pregnant, lactating, of childbearing potential, or planning to become pregnant

10) Patients with a history of hypersensitivity to the study drug

11) Patients who are determined by the investigator to be not suitable for other reasons

Revised Text

4. Subjects

(2) Exclusion criteria

Patients who meet any of the following criteria will not be included in the study:

1) Patients with type 1 DM

2) Patients with a history of severe ketosis, diabetic coma or precoma within the past 6 months

3) Patients with severe infection, in a perioperative condition or with serious trauma

4) Patients with severe renal impairment (glomerular filtration rate: eGFR < 30 mL/min or dialysis patients)

5) Patients who developed myocardial infarction, angina pectoris or underwent percutaneous transluminal coronary angioplasty or bypass surgery, developed cerebral infarction, cerebral hemorrhage, subarachnoid hemorrhage or transient ischemic attack within 3 months prior to the start of the study

6) Patients with moderate or more severe heart failure (NYHA functional class III or IV)

7) Patients who are on DPP-4 inhibitors or **GLP-1 analogues** at the time of study initiation

8) Patients who are on insulin at the time of study initiation

9) Patients who are pregnant, lactating, of childbearing potential, or planning to become pregnant

10) Patients with a history of hypersensitivity to the study drug

11) Patients who are determined by the investigator to be not suitable for other reasons **such as malignancies**

Rationale for Amendment

GLP-1 analogues have pharmacological properties similar to those of DPP-4 inhibitors.

A patient with a malignancy may suffer a disadvantage.

*2. The concomitant use of sitagliptin and α-GI*

Previous Text

6. Method of the Study

(6) Rules for concomitant medications (therapies)

1) Prohibited concomitant medications

Group A: DPP-4 inhibitors except sitagliptin, α-GI drug, glinide and insulin

Group B: DPP-4 inhibitors, and insulin

Revised Text

6. Method of Study

(6) Rules for concomitant medications (therapies)

1) Prohibited concomitant medications

Group A: DPP-4 inhibitors except sitagliptin, α-GI drug*****, glinide, and insulin

Group B: DPP-4 inhibitors, and insulin

*** The concomitant use of sitagliptin and α-GI is under discussion for approval. After approval, concomitant use can be used.**

Rationale for Amendment

The concomitant use of sitagliptin and α-GI is under discussion for approval. After approval, the concomitant use can be used.

*3.. Combination therapy with sitagliptin and alpha-GI drug.*

Previous Text

(2) Outline of the study

| Target plasma glucose control level: HbA1c (JDS) < 5.8% or fasting plasma glucose < 110 mg/dL | | | | |  |
| --- | --- | --- | --- | --- | --- |
|  | | | | | |
| Group A: Sitagliptin group | | | | | |
|  | | | | Not achieving the target: Increase the dose of the concomitant medication, etc. | |
|  | | | Not achieving the target: Increase the dose of sitagliptin to 100 mg | | |
|  | | Sitagliptin 50 mg^*1,*2^ | | | |
| 5.8% ≤ HbA1c (JDS) < 9.0%   1. Diet/exercise therapy only 2. Diet/exercise therapy + oral antidiabetic drugs (receiving treatment with antidiabetic drugs excluding DPP-4 inhibitors) | ^*1^ When patients who are on α-GI or glinide are assigned to Group A, α-GI or glinide is discontinued and switched to sitagliptin.  ^*2^ The specific dosage regimen is described in Section 6. Method of the Study, (4) Treatment schedule. | | | | |
| Group B: Conventional group | | Oral antidiabetic drugs excluding DPP-4 inhibitors^*2^ | | | |

-3M 0M 3M 6M 12M 24M

Revised Text

(2) Outline of the study

| Target plasma glucose control level: HbA1c (JDS) < 5.8% or fasting plasma glucose < 110 mg/dL | | | | |  |
| --- | --- | --- | --- | --- | --- |
|  | | | | | |
| Group A: Sitagliptin group | | | | | |
|  | | | | Not achieving the target: Increase the dose of the concomitant medication, etc. | |
|  | | | Not achieving the target: Increase the dose of sitagliptin to 100 mg | | |
|  | | Sitagliptin 50 mg^*1,*2^ | | | |
| 5.8% ≤ HbA1c (JDS) < 9.0%  (1)Diet/exercise therapy only  (2) Diet/exercise therapy + oral antidiabetic drugs (receiving treatment with antidiabetic drugs excluding DPP-4 inhibitors) | ^*1^ When patients who are on α-GI or glinide are assigned to Group A, α-GI or glinide is discontinued and switched to sitagliptin.*** The doses of the antidiabetic drugs used may be reduced at the start of the study.**  ^*2^ The specific dosage regimen is described in Section 6. Method of the Study, (4) Treatment schedule. | | | | |
| Group B: Conventional group | | Oral antidiabetic drugs excluding DPP-4 inhibitors^*2^ | | | |

-3M 0M 3M 6M 12M 24M

*** The concomitant use of sitagliptin and α-GI is under discussion for approval. After approval, concomitant use can be used.**

Rationale for Amendment

Combination therapy with sitagliptin and alpha-GI drug is under discussion for approval.

*4. “Use of HMG-CoA Reductase Inhibitor“ was added to random allocation factors*

Previous Text

14. Data Tabulations and Statistical Analysis Methods

(2) Analysis methods

3) Analysis of the primary endpoint (IMT)

The primary objective of this study is to evaluate whether or not the percent change in IMT at Month 24, the primary endpoint, significantly decreases in the sitagliptin group compared to that in the conventional group. A null hypothesis that the percent change in IMT between the two groups is equivalent in the primary analysis will be performed using the analysis of covariance (ANCOVA). At that time, covariates will be randomization factors (age, gender, blood pressure and baseline HbA1c and IMT). In addition, as sensitivity analysis, the time-course of IMT in each group will be presented, and longitudinal data will be analyzed using a linear mixed effects model to confirm that the results are similar to those of the ANCOVA. The significance level will be 0.05 for a two-sided test, and two-sided 95% confidence intervals will be calculated.

Revised Text

14. Data Tabulations and Statistical Analysis Methods

(2) Analysis methods

3) Analysis of the primary endpoint (IMT)

The primary objective of this study is to evaluate whether or not the percent change in IMT at Month 24, the primary endpoint significantly decreases in the sitagliptin group compared with that in the conventional group. A null hypothesis that the percent change in IMT between the two groups is equivalent in the primary analysis will be performed using analysis of covariance (ANCOVA). At that time, covariates will be randomization factors (**statin use**, age, gender, blood pressure, and baseline HbA1c and IMT). In addition, as sensitivity analysis, the time-course of IMT in each group will be presented, and longitudinal data will be analyzed using a linear mixed effects model to confirm that the results are similar to those of the ANCOVA. The significance level will be 0.05 for a two-sided test, and two-sided 95% confidence intervals will be calculated.

Rationale for Amendment

HMG-CoA Reductase Inhibition is known to reduce carotid IMT.

Ref Nohara R,, et al. Effect of long-term intensive lipid-lowering therapy with rosuvastatin on progression of

carotid intima-media thickness--Justification for Atherosclerosis Regression Treatment (JART) extension study.

Circ J. 2013;77:1526-33.

*5. “Use of GLP-1 analogue” was prohibited in the sitagliptin group.*

Previous Text

6. Method of the Study

(6) Rules for concomitant medications (therapies)

1) Prohibited concomitant medications

Group A: DPP-4 inhibitors except sitagliptin, glinide and insulin

Group B: DPP-4 inhibitors and insulin

Revised Text

6. Method of Study

(6) Rules for concomitant medications (therapies)

1) Prohibited concomitant medications

Group A: DPP-4 inhibitors except sitagliptin, **GLP-1 analogues**, glinide, and insulin

Group B: DPP-4 inhibitors, **GLP-1 analogues** and insulin

Rationale for Amendment

GLP-1 analogues have pharmacologiocal properties similar to those of DPP-4 inhibitors.

*6. Roche Diagnostics K.K. and Fujirebio Inc. (23.Study Organization (8) Joint study sites)*

New Text

23. Study Organization

(8) Joint study sites

Measurement of high molecular weight adiponectin

Fujirebio Inc.

FR Building, 2-62-5 Nihonbashihamacho, Chuo-ku, Tokyo 103-0007

Measurement of NT-proBNP

Roche Diagnostics K.K. 2-6-1 Shiba, Minato-ku, Tokyo 105-0014

Rationale for Amendment

Joint study sites and their addresses added to clarify their roles.

*7. Yasunori Sato joined as a member of Statistical Analysis Committee (23.Study Organization (7) Statistical Analysis Committee).*

New Text

23. Study Organization

(7) Statistical Analysis Committee

Yasunori Sato, Instructor, Department of Global Clinical Research, Graduate School of Medicine, Chiba University.

Rationale for Amendment

Yasunori Sato joined as a member of Statistical Analysis Committee (23.Study Organization (7) Statistical Analysis Committee).

*8. Hirotsugu Yamada joined as a member of working group of echocardiography (23.Study Organization (5) Working Group).*

New Text

23. Study Organization

(5) Working Group

| FMD (representative) | Hiroshima University | Department of Cardiovascular Physiology and Medicine | Associate Professor | Yukihito Higashi |
| --- | --- | --- | --- | --- |
| FMD | Shimane University | Postgraduate Clinical Training Center | Associate Professor | Yutaka Ishibashi |
| FMD | Osaka City University | Department of Cardiology | Instructor | Kenei Shimada |
| PWV/CAVI/AI | Tokyo Medical University | Department of Cardiology | Professor | Hirofumi Tomiyama |
| IMT | University of Tsukuba | Department of Clinical Laboratory Medicine, Faculty of Medicine | Instructor | Tomoko Ishizu |
| **Echocardiography** | **Tokushima University** | **Department of Cardiovascular Medicine/Ultrasound Examination Center** | **Instructor** | **Hirotsugu Yamada** |

Rationale for Amendment

Hirotsugu Yamada joined as a member of working group on echocardiography (23.Study Organization (5) Working Group).

*9. Schedule for the study*

Previous Text

6. Method of the Study

(2) Outline of the study

| Target plasma glucose control level: HbA1c (JDS) < 5.8% or fasting plasma glucose < 110 mg/dL | | | | |  |
| --- | --- | --- | --- | --- | --- |
|  | | | | | |
| Group A: Sitagliptin group | | | | | |
|  | | | | Not achieving the target: Increase the dose of the concomitant medication, etc. | |
|  | | | Not achieving the target: Increase the dose of sitagliptin to 100 mg | | |
|  | | Sitagliptin 50 mg^*1,*2^ | | | |
| 5.8% ≤ HbA1c (JDS) < 9.0%   1. Diet/exercise therapy only 2. Diet/exercise therapy + oral antidiabetic drugs (receiving treatment with antidiabetic drugs excluding DPP-4 inhibitors) | ^*1^ When patients who are on α-GI or glinide are assigned to Group A, α-GI or glinide is discontinued and switched to sitagliptin.* The dosage of the antidiabetic drugs that are used may be reduced at the start of the study.  ^*2^ The specific dosage regimen is described in Section 6. Method of the Study, (4) Treatment schedule. | | | | |
| Group B: Conventional group | | Oral antidiabetic drugs excluding DPP-4 inhibitors^*2^ | | | |

**-3M** 0M 3M 6M 12M 24M

* The concomitant use of sitagliptin and α-GI is under discussion for approval. After approval, the concomitant use can be used.

(4) Treatment schedule

1) Before the start of the study

After selecting the subjects from past medical records **for more than three months**, the investigator will give an explanation on the contents of the study to the subjects using the informed consent document and receive written consent from them.

The subjects will be assigned to either the non-drug therapy or drug therapy group according to the treatment method at the time of enrollment. Of the background characteristics of the subjects in each group, age, gender, blood pressure, and baseline HbA1c and IMT levels as prognosis predictors, computerized randomization will be performed using a minimization method so that the prognosis predictors will be equally assigned to Group A: sitagliptin group and Group B: conventional group.

Revised Text

Deleted.

6. Method of Study

(2) Outline of the study

| Target plasma glucose control level: HbA1c (JDS) < 5.8% or fasting plasma glucose < 110 mg/dL | | | | |  |
| --- | --- | --- | --- | --- | --- |
|  | | | | | |
| Group A: Sitagliptin group | | | | | |
|  | | | | Not achieving the target: Increase the dose of the concomitant medication, etc. | |
|  | | | Not achieving the target: Increase the dose of sitagliptin to 100 mg | | |
|  | | Sitagliptin 50 mg^*1,*2^ | | | |
| 5.8% ≤ HbA1c (JDS) < 9.0%   1. Diet/exercise therapy only 2. Diet/exercise therapy + oral antidiabetic drugs (receiving treatment with antidiabetic drugs excluding DPP-4 inhibitors) | ^*1^ When patients who are on α-GI or glinide are assigned to Group A, α-GI or glinide is discontinued and switched to sitagliptin.* The dosage of the antidiabetic drugs that are used may be reduced at the start of the study.  ^*2^ The specific dosage regimen is described in Section 6. Method of the Study, (4) Treatment schedule. | | | | |
| Group B: Conventional group | | Oral antidiabetic drugs excluding DPP-4 inhibitors^*2^ | | | |

0M 3M 6M 12M 24M

* The concomitant use of sitagliptin and α-GI is under discussion for approval. After approval, concomitant use can be used.

(4) Treatment schedule

1) Before the start of the study

After selecting the subjects from past medical records, the investigator will give an explanation of the content of the study to the subjects using the informed consent document and receive written consent from them.

The subjects will be assigned to either the non-drug therapy or drug therapy group according to the treatment method at the time of enrollment. Using the background characteristics of the subjects in each group (**statin use**, age, gender, blood pressure, and baseline HbA1c and IMT levels) as prognostic predictors, computerized randomization will be performed using a minimization method so that the predictors will be equally assigned to Group A: sitagliptin group and Group B: conventional group.

Rationale for Amendment

The patients were already treated and observed for more than three months, which is described in “Inclusion criteria”; “statin use” added as a prognosis predictor.

*10. Amylase and other explanations were added in “7. Endpoints and 8. Observations and Tests*

Previous Text

7. Endpoints

(2) Secondary endpoints

3) Observed values at baseline, Months 3, 6, 12 and 24, and changes/percent

changes from baseline for the following laboratory tests:

HbA1c, fasting plasma glucose, insulin, serum lipid (TC, HDL-C and TG),

serum creatinine, urinary albumin excretion (corrected by creatinine), eGFR

(converted value), HOMA-β, HOMA-R (converted value) and cystatin C

8. Observations and Tests

(7) Blood biochemistry: Baseline and Months 3*, 6*, 12 and 24 (*Moths 3 and 6 are optional except for HbA1c) AST, ALT, LDH, serum creatinine, BUN, uric acid, Na, K, Cl, TC, HDL-C, TG, plasma glucose, HbA1c, insulin, cystatin C.

Revised Text

7. Endpoints

(2) Secondary endpoints

3) Observed values at baseline, Months 3, 6, 12 and 24, and changes/percent

changes from baseline for the following laboratory tests:

HbA1c, fasting plasma glucose, insulin, serum lipid (TC, HDL-C and TG),

serum creatinine, urinary albumin excretion (corrected for creatinine excretion rate), eGFR (converted value), HOMA-β **(converted value)**, HOMA-R (converted value) and cystatin C.

8. Observations and Tests

(7) Blood biochemistry: Baseline and Months 3*, 6*, 12 and 24 (*Months 3 and 6 are optional except for HbA1c) AST, ALT, LDH, serum creatinine, BUN, uric acid, Na, K, Cl, TC, HDL-C, TG, plasma glucose, HbA1c, insulin, cystatin C **(at the time of decreased renal function)** and **amylase.**

Rationale for Amendment

The measurement of amylase added to check pancreatitis.

The explanations added.

*11. Appendix 5) Manual for procedures for echocardiography added.*

New Text

Appendix 5) Manual for procedures for echocardiography

Rationale for Amendment

Manual for procedures for echocardiography added.

***Ver. 3.0 documented on May 1, 2012***

*1. Combination therapy with sitagliptin and alpha-GI drug*

Previous Text

6. Method of the Study

(2) Outline of the study

| Target plasma glucose control level: HbA1c (JDS) < 5.8% or fasting plasma glucose < 110 mg/dL | | | | |  |
| --- | --- | --- | --- | --- | --- |
|  | | | | | |
| Group A: Sitagliptin group | | | | | |
|  | | | | Not achieving the target: Increase the dose of the concomitant medication, etc. | |
|  | | | Not achieving the target: Increase the dose of sitagliptin to 100 mg | | |
|  | | Sitagliptin 50 mg^*1,*2^ | | | |
| 5.8% ≤ HbA1c (JDS) < 9.0%   1. Diet/exercise therapy only 2. Diet/exercise therapy + oral antidiabetic drugs (receiving treatment with antidiabetic drugs excluding DPP-4 inhibitors) | ^*1^ When patients who are on **α-GI** or glinide are assigned to Group A, glinide is discontinued and switched to sitagliptin.***** The dosage of the antidiabetic drugs that are used may be reduced at the start of the study.  ^*2^ The specific dosage regimen is described in Section 6. Method of the Study, (4) Treatment schedule. | | | | |
| Group B: Conventional group | | Oral antidiabetic drugs excluding DPP-4 inhibitors^*2^ | | | |

0M 3M 6M 12M 24M

*** The concomitant use of sitagliptin and α-GI is under discussion for approval. After approval, the concomitant use can be used.**

6. Method of the Study

(6) Rules for concomitant medications (therapies)

1) Prohibited concomitant medications

Group A: DPP-4 inhibitors except sitagliptin, GLP-1 analogues, **alpha-GI drug**, glinide and insulin

Group B: DPP-4 inhibitors, GLP-1 analogues and insulin

Revised Text

Deleted

6. Method of the Study

(2) Outline of the study

| Target plasma glucose control level: HbA1c (JDS) < 5.8% or fasting plasma glucose < 110 mg/dL | | | | |  |
| --- | --- | --- | --- | --- | --- |
|  | | | | | |
| Group A: Sitagliptin group | | | | | |
|  | | | | Not achieving the target: Increase the dose of the concomitant medication, etc. | |
|  | | | Not achieving the target: Increase the dose of sitagliptin to 100 mg | | |
|  | | Sitagliptin 50 mg^*1,*2^ | | | |
| 5.8% ≤ HbA1c (JDS) < 9.0%   1. Diet/exercise therapy only 2. Diet/exercise therapy + oral antidiabetic drugs (receiving treatment with antidiabetic drugs excluding DPP-4 inhibitors) | ^*1^ When patients who are on glinide are assigned to Group A, glinide is discontinued and switched to sitagliptin. The dosage of the antidiabetic drugs that are used may be reduced at the start of the study.  ^*2^ The specific dosage regimen is described in Section 6. Method of the Study, (4) Treatment schedule. | | | | |
| Group B: Conventional group | | Oral antidiabetic drugs excluding DPP-4 inhibitors^*2^ | | | |

0M 3M 6M 12M 24M

6. Method of the Study

(6) Rules for concomitant medications (therapies)

1) Prohibited concomitant medications

Group A: DPP-4 inhibitors except sitagliptin, GLP-1 analogues, glinide and insulin

Group B: DPP-4 inhibitors, GLP-1 analogues and insulin

Rationale for Amendment

Combination therapy with sitagliptin and an alpha-GI drug is available by approval of Ministry of Health, Labour and Welfare in Japan.

*2. The primary endpoint was defined from “amount and rate of change in common carotid artery” to “rate of change in common carotid artery”. “amount of change in common carotid artery” was defined in secondary endpoint.*

Previous Text

7. Endpoints

(1) Primary endpoint

**Amount of change and** percent change in mean common carotid artery (CCA)-IMT at Month 24 from baseline as measured by carotid artery echography

(2) Secondary endpoints

1) Observed IMT values at baseline, Month 12 and Month 24 as measured by carotid artery echography (CCA, carotid bulbs and internal carotid artery [ICA]) and the change/percent change from baseline (excluding mean CCA-IMT at Month 24 from baseline)

Maximum IMT, plaque area, and grey scale value

Revised Text

7. Endpoints

(1) Primary endpoint

Percent change in mean common carotid artery (CCA)-IMT at Month 24 from baseline as measured by carotid artery echography

(2) Secondary endpoints

1) Observed IMT values at baseline, Month 12 and Month 24 as measured by carotid artery echography (CCA, carotid bulbs and internal carotid artery [ICA]) and the change/percent change from baseline (excluding **the percent change** in mean CCA-IMT at Month 24 from baseline)

**Mean IMT**, maximum IMT, plaque area, and grey scale value

Rationale for Amendment

Because the amount of change in mean CCA-IMT correlates very strongly with the percentage change in mean CCA-IMT, the primary endpoint is defined only as a percentage change in mean CCA-IMT at Month 24 from the baseline. The amount of change in mean CCA-IMT at Month 24 from baseline and mean IMT were defined as secondary endpoints.

*3. Interim analysis is performed at one year after the registration of the final patient.*

New Text

Added.

**14. Data Tabulations and Statistical Analysis Methods**

**(2) Analysis methods**

**6) Interim analysis**

**Considering the current situation that continuing treatment for a long time without the use of DPP-4 inhibitors is becoming difficult and the vascular protection effects of DPP-4 inhibitors are starting to be reported, it is necessary to evaluate the results of this study as soon as possible from the viewpoint of welfare of patients. Consequently, an interim analysis will be performed one year after the end of patient enrollment. The details are separately described in the interim analysis plan.**

Rationale for Amendment

Clinically DPP-4 inhibitors are prevalent after launch and approximately half of T2DM patients use one in Japan. Basic researches ^1, 2)^ and clinical meta-analyses ^3, 4)^ found that DPP-4 inhibitors have potent beneficial effects on cardiovascular diseases. Additionally, DPP-4 inhibitors have been reported to prevent the progression of carotid atherosclerosis in small numbers in domestic and international scientific meetings. Therefore, it is difficult to enroll patients who meet the criteria, and furthermore the patients in the conventional treatment group may suffer a disadvantage from the treatment for two years. In view of these circumstances, the steering committee decided to perform an interim analysis one year after the enrollment of the final patient.

Ref

1) Ta NN, et al. DPP-4 (CD26) inhibitor alogliptin inhibits atherosclerosis in diabetic apolipoprotein E-deficient mice. J Cardiovasc Pharmacol. 2011 Aug;58(2):157-66.

2) Matsubara J, et al. A dipeptidyl peptidase-4 inhibitor, des-fluoro-sitagliptin, improves endothelial function and reduces atherosclerotic lesion formation in apolipoprotein E-deficient mice.J Am Coll Cardiol. 2012 Jan 17;59(3):265-76.

3) Fadini GP, et al. The oral dipeptidyl peptidase-4 inhibitor sitagliptin increases circulating endothelial progenitor cells in patients with type 2 diabetes: possible role of stromal-derived factor-1alpha. Diabetes Care. 2010 Jul;33(7):1607-9.

4) Johansen OE, et al. Cardiovascular safety with linagliptin in patients with type 2 diabetes mellitus: a pre-specified, prospective, and adjudicated meta-analysis of a phase 3 programme. Cardiovasc Diabetol. 2012 Jan 10;11:3.

5) Mosenzon O, et al. Potential cardiovascular effects of dipeptidyl peptidase-4 inhibitors in patients with type 2 diabetes: current evidence and ongoing trials. Eur Heart J Suppl 2012; 14 (B): B22-B29.

*5. Relocation*

Previous Text

23. Study Organization

(3) Independent Efficacy and Safety Evaluation Committee

| Chairperson/Neurology | Kawasaki Medical School | Department of Nephrology and Hypertension | Professor | Naoki Kashihara |
| --- | --- | --- | --- | --- |
| Cardiovascular | **Hiroshima City Hiroshima Citizens Hospital** | **Department of Cardiology** | Director | Masaharu Ishihara |
| Diabetes | Tokyo Medical University | Department of Diabetes, Endocrinology, Metabolism and Rheumatology | Professor | Masato Odawara |
| Diabetes | University of the Ryukyus | Second Department of Medicine, Division of Endocrinology, Diabetes and Metabolism, Hematology, Rheumatology | Professor | Hiroaki Masuzaki |
| Stroke | Osaka University | Department of Neurology | Associate Professor | Kazuo Kitagawa |
| Gastroenterology | Osaka University | Department of Gastroenterology | Instructor | Masahiko Tsujii |

(5) Working Group

| FMD (representative) | Hiroshima University | **Department of Cardiovascular Physiology and Medicine** | **Associate Professor** | Yukihito Higashi |
| --- | --- | --- | --- | --- |
| FMD | Shimane University | **Postgraduate Clinical Training Center** | **Associate Professor** | Yutaka Ishibashi |
| FMD | Osaka City University | **Department of Cardiology** | **Instructor** | Kenei Shimada |
| PWV/CAVI/AI | Tokyo Medical University | Department of Cardiology | Professor | Hirofumi Tomiyama |
| IMT | University of Tsukuba | Department of Clinical Laboratory Medicine, Faculty of Medicine | Instructor | Tomoko Ishizu |
| Echocardiography | Tokushima University | Department of Cardiovascular Medicine/Ultrasound Examination Center | Instructor | Hirotsugu Yamada |

Revised Text

23. Study Organization

(3) Independent Efficacy and Safety Evaluation Committee

| Chairperson/Neurology | Kawasaki Medical School | Department of Nephrology and Hypertension | Professor | Naoki Kashihara |
| --- | --- | --- | --- | --- |
| Cardiovascular | **National Cerebral and Cardiovascular Center** | **Department of Clinical Medicine and Development** | Director | Masaharu Ishihara |
| Diabetes | Tokyo Medical University | Department of Diabetes, Endocrinology, Metabolism and Rheumatology | Professor | Masato Odawara |
| Diabetes | University of the Ryukyus | Second Department of Medicine, Division of Endocrinology, Diabetes and Metabolism, Hematology, Rheumatology | Professor | Hiroaki Masuzaki |
| Stroke | Osaka University | Department of Neurology | Associate Professor | Kazuo Kitagawa |
| Gastroenterology | Osaka University | Department of Gastroenterology | Instructor | Masahiko Tsujii |

(5) Working Group

| FMD (representative) | Hiroshima University | **Research Institute for Radiation Biology and Medicine** | **Professor** | Yukihito Higashi |
| --- | --- | --- | --- | --- |
| FMD | Shimane University | **Department of General Medicine** | **Professor** | Yutaka Ishibashi |
| FMD | Osaka City University | **Department of Medical Education and General Practice** | **Instructor** | Kenei Shimada |
| PWV/CAVI/AI | Tokyo Medical University | Department of Cardiology | Professor | Hirofumi Tomiyama |
| IMT | University of Tsukuba | Department of Clinical Laboratory Medicine and Cardiology, Faculty of Medicine | Instructor | Tomoko Ishizu |
| Echocardiography | Tokushima University | Department of Cardiovascular Medicine/Ultrasound Examination Center | Instructor | Hirotsugu Yamada |

Rationale for Amendment

Titles and Institution changed.

***Ver. 4.0 documented on June 19, 2012***

*1. Target Sample Size changed in “15. Target Sample Size and Rationale for Determination”.*

Previous Text

15. Target Sample Size and Rationale for Determination

(1) Target sample size

**1200** patients (**600** patients in each group)

(2) Rationale for determination

Yamasaki reported that et al. reported that there is significant relationship between increased rate of carotid IMT and mean HbA1c in Japanese T2DM and suggested that the glycemic control can influence the progression of atherosclerosis. ^1)^ Assuming that annual increase in carotid IMT is**＋0.005 ± 0.06 mm** in patients with mean HbA1c of 7.0% and **-0.005 ± 0.06 mm** (mean ± standard deviation) in patients with mean HbA1c of 6.0% from the report^1)^, the minimum number of patients necessary for detecting a significant difference between the two groups under conditions, i.e., a hazard ratio of 5% and power of 80%, is **1134** patients in total or **567** patients in each group. If there is a difference of 1% HbA1c between the sitagliptin group and the conventional group, the target number of patients to be enrolled is 500 patients in total or 250 patients in each group, assuming a dropout of approximately 5%.

^1)^　Yamasaki Y *et al*; Carotid Intima-Media Thickness in Japanese Type 2 Diabetic Subjects. *Diabetes Care* 2000: 9, 1310-1315

Revised Text

15. Target Sample Size and Rationale for Determination

(1) Target sample size

**500** patients (**250** patients in each group)

(2) Rationale for determination

**In CHICAGO study^1)^, in which the effect of thiazolidines and SU on CCA-IMT was investigated in T2DM patients, there was no significant difference in HbA1c up to Week 48 between the two groups, but the mean change in IMT was -0.001 mm in the pioglitazone group and +0.012 mm in the glimepiride group; thus, it was reported that the progression of CCA-IMT was significantly inhibited in the pioglitazone group.**

**At present, no study of the effect of DPP-4 inhibitors on CCA-IMT has been reported.** **However, assuming that sitagliptin has an effect of inhibiting progression of CCA thickness equivalent to that of pioglitazone, and the change in CCA-IMT at Week 48 would be -0.001 ± 0.05 mm in the sitagliptin group and +0.012 ± 0.05 mm in the conventional group,** the minimum number of patients necessary for detecting a significant difference between the two groups under conditions, i.e., a hazard ratio of 5% and power of 80%, is **464** patients in total or **232** patients in each group.

**In addition, when the change in CHICAGO study is converted to a per cent change, and the per cent change in CCA-IMT is assumed to be -0.130 ± 6.5% in the sitagliptin group and 1.540 ± 6.5% in the conventional group,** the minimum number of patients necessary for detecting a significant difference between the two groups under conditions, i.e., a hazard ratio of 5% and power of 80%, is **476** patients in total or **238** patients in each group.

Assuming a dropout of approximately 5%, the target number of patients to be enrolled is **500** patients in total or **250** patients in each group.

**26. List of References/ Literature**

**^1)^ Mazzone T *et al*; Effect of Pioglitazone Compared With Glimepiride on Carotid Intima-Media Thickness in Type 2 Diabetes. *JAMA* 2006: 296, 2572-2581**

Rationale for Amendment

It is difficult to determine accurately the necessary sample size for testing a new drug. Owing to their common use in Japan, it is difficult not to use DPP-4 inhibitors clinically. Recent basic researches and meta-analyses suggested that DPP-4 inhibitors have anti-atherosclerosis properties. Besides, it has been reported that DPP-4 inhibitors reduced carotid IMT in small numbers of the patients in a scientific meeting. Based on the accumulating evidence described above, the sample size was recalculated assuming that DPP-4 inhibitors have similar protective effects to thiazolidine derivatives, which have been reported to prevent carotid IMT (based on CHICAGO study)^1^.

*2. The end of implementation period extended from “June 30 2014” to “Sept 30 2014”.*

Previous Text

13. Period of the Study

Date of approval of the ethics review committee to **June 30, 2014**

Treatment observation period: **June 30**, **2014** (closing date of enrollment: September 30, 2012)

Study period: **June 30, 2014** (publication of analysis and study results)

Revised Text

13. Period of the Study

Date of approval of the ethics review committee to **September 30, 2014**

Treatment observation period: **September 30**, **2014** (closing date of enrollment: September 30, 2012)

Study period: **September 30, 2014** (publication of analysis and study results)

Rationale for Amendment

The period of the study is extended to September 30, 2014.

*3. Takanori Yasu joined as a member of “Independent Data Monitoring Board” (23.Study Organization (4) Independent Data Monitoring Board).*

New Text

23. Study Organization

(4) Independent Data Monitoring Board

| Chairperson | Tokyo Medical University | Department of Cardiology | Professor | Akira Yamashina |
| --- | --- | --- | --- | --- |
|  | Jikei University School of Medicine | Division of Molecular Epidemiology | Professor | Mitsuyoshi Urashima |
|  | **Dokkyo Medical University** | **Department of Cardiovascular Medicine, Nikko Medical Center** | **Professor** | **Takanori Yasu** |

Rationale for Amendment

Takanori Yasu joined as a member of “Independent Data Monitoring Board” (23.Study Organization (4) Independent Data Monitoring Board).

***Ver. 4.1 documented on February 22, 2013***

*1. One of Research secretariats (Dr. Kazuhisa Kodama) was deleted due to the relocation.*

Previous Text

23. Study Organization

(9) Study secretariat

Jun-ichi Oyama, **Kazuhisa Kodama**

Department of Cardiovascular Medicine, Saga University Faculty of Medicine

5-1-1 Nabeshima, Saga, 849-8501

Revised text

Removed.

23. Study Organization

(9) Study secretariat

Jun-ichi Oyama

Department of Cardiovascular Medicine, Saga University Faculty of Medicine

5-1-1 Nabeshima, Saga, 849-8501

Rationale for Amendment

Dr. Kazuhisa Kodama is no longer with Saga University owing to his relocation.

***Ver. 5.0 documented on April 1, 2014***

*1. The end of implementation period extended from “Sept. 30 2014” to “Sept. 30 2015”.*

Previous Text

13. Period of the Study

Date of approval of the ethics review committee to **September 30, 2014**

Treatment observation period: **September 30**, **2014** (closing date of enrollment: September 30, 2012)

Study period: **September 30, 2014** (publication of analysis and study results)

Revised Text

13. Period of the Study

Date of approval of the ethics review committee to **September 30, 2015**

Treatment observation period: **December 31, 2014** (closing date of enrollment: September 30, 2012)

Study period: **September 30, 2015** (publication of analysis and study results)

Rationale for Amendment

The period of the study is extended to September 30, 2015.

*2. Audit was stipulated.*

Previous Text

None

New Text

21. Audits

Audits are intended to determine whether or not this study is properly conducted and ensure the reliability of the data. The study organization will contract out audits of the study to an external party. When performing audits, records revealing the identities of the patients and confidential medical information should be kept to protect privacy. The investigators and study sites should provide records on the study for direct access upon request and collaborate in this at the time of audits and investigations by the ethics review committee.

Rationale for Amendment

Audit was stipulated.

*3. Relocation*

Previous Text

23. Study Organization

(2) Steering committee

| Cardiovascular | Hokkaido University | Department of Cardiovascular Medicine | Professor | Hiroyuki Tsutsui |
| --- | --- | --- | --- | --- |
| Cardiovascular | Akita University | Department of Cardiovascular and Respiratory Medicine | Professor | Hiroshi Ito |
| Cardiovascular | Dokkyo Medical University | Department of Cardiovascular Medicine | Professor | Teruo Inoue |
| Cardiovascular | Showa University | Department of Diabetes, Metabolism, and Endocrinology | Professor | Tsutomu Hirano |
| Cardiovascular | Juntendo University | Department of Cardiovascular Medicine | Professor | Hiroyuki Daida |
| Cardiovascular | Mie University | Department of Cardiology and Nephrology | Professor | Masaaki Ito |
| Cardiovascular | National Cerebral and Cardiovascular Center | Department of Clinical Medicine and Development | Director | Masafumi Kitakaze |
| Cardiovascular | Tokushima University | Department of Cardiovascular Medicine | Professor | Masataka Sata |
| Cardiovascular | Nagasaki University | Department of Cardiovascular Medicine | Professor | Koji Maemura |
| Hypertension | University of the Ryukyus Hospital | Clinical Research Support Center | Professor | Shinichiro Ueda |
| Diabetes | Tokushima University | Diabetes Therapeutics and Research Center | Professor | Munehide Matsuhisa |
| Diabetes | Kawasaki Medical School | Department of Internal Medicine | Professor | Kohei Kaku |
| Metabolism | Yokohama City University | Department of Endocrinology and Metabolism | Professor | Yasuo Terauchi |
| Neurology | Tokyo Women’s Medical University | Department of Medicine, Kidney Center | Professor | Kosaku Nitta |
| Neurology | Nippon Medical School | Department of Nephrology | Professor | **Yasuhiko Iino** |

(3) Independent Efficacy and Safety Evaluation Committee

| Chairperson/Neurology | Kawasaki Medical School | Department of Nephrology and Hypertension | Professor | Naoki Kashihara |
| --- | --- | --- | --- | --- |
| Cardiovascular | National Cerebral and Cardiovascular Center | Department of Clinical Medicine and Development | Director | Masaharu Ishihara |
| Diabetes | Tokyo Medical University | Department of Diabetes, Endocrinology, Metabolism and Rheumatology | Professor | Masato Odawara |
| Diabetes | University of the Ryukyus | Second Department of Medicine, Division of Endocrinology, Diabetes and Metabolism, Hematology, Rheumatology | Professor | Hiroaki Masuzaki |
| Stroke | **Osaka University** | Department of Neurology | **Associate Professor** | Kazuo Kitagawa |
| Gastroenterology | Osaka University | Department of Gastroenterology | **Instructor** | Masahiko Tsujii |

(4) Independent Data Monitoring Board

| Chairperson | Tokyo Medical University | Department of Cardiology | Professor | Akira Yamashina |
| --- | --- | --- | --- | --- |
|  | Jikei University School of Medicine | Division of Molecular Epidemiology | **Associate Professor** | Mitsuyoshi Urashima |
|  | Dokkyo Medical University | Department of Cardiovascular Medicine, Nikko Medical Center | Professor | Takanori Yasu |

(5) Working Group

| FMD (representative) | Hiroshima University | Research Institute for Radiation Biology and Medicine | Professor | Yukihito Higashi |
| --- | --- | --- | --- | --- |
| FMD | Shimane University | Department of General Medicine | Professor | Yutaka Ishibashi |
| FMD | Osaka City University | **Department of Medical Education and General Practice** | **Instructor** | Kenei Shimada |
| PWV/CAVI/AI | Tokyo Medical University | Department of Cardiology | Professor | Hirofumi Tomiyama |
| IMT | University of Tsukuba | **Department of Clinical Laboratory Medicine and Cardiology, Faculty of Medicine** | Instructor | Tomoko Ishizu |
| Echocardiography | Tokushima University | Department of Cardiovascular Medicine/Ultrasound Examination Center | Instructor | Hirotsugu Yamada |

(6) Measurement laboratories

Centralized measurement laboratories for IMT

Department of Clinical Laboratory Medicine and **Cardiovascular Division**, University of Tsukuba

Revised Text

23. Study Organization

(2) Steering committee

| Cardiovascular | Hokkaido University | Department of Cardiovascular Medicine | Professor | Hiroyuki Tsutsui |
| --- | --- | --- | --- | --- |
| Cardiovascular | Akita University | Department of Cardiovascular and Respiratory Medicine | Professor | Hiroshi Ito |
| Cardiovascular | Dokkyo Medical University | Department of Cardiovascular Medicine | Professor | Teruo Inoue |
| Cardiovascular | Showa University | Department of Diabetes, Metabolism, and Endocrinology | Professor | Tsutomu Hirano |
| Cardiovascular | Juntendo University | Department of Cardiovascular Medicine | Professor | Hiroyuki Daida |
| Cardiovascular | Mie University | Department of Cardiology and Nephrology | Professor | Masaaki Ito |
| Cardiovascular | National Cerebral and Cardiovascular Center | Department of Clinical Medicine and Development | Director | Masafumi Kitakaze |
| Cardiovascular | Tokushima University | Department of Cardiovascular Medicine | Professor | Masataka Sata |
| Cardiovascular | Nagasaki University | Department of Cardiovascular Medicine | Professor | Koji Maemura |
| Hypertension | University of the Ryukyus Hospital | Clinical Research Support Center | Professor | Shinichiro Ueda |
| Diabetes | Tokushima University | Diabetes Therapeutics and Research Center | Professor | Munehide Matsuhisa |
| Diabetes | Kawasaki Medical School | Department of Internal Medicine | Professor | Kohei Kaku |
| Metabolism | Yokohama City University | Department of Endocrinology and Metabolism | Professor | Yasuo Terauchi |
| Neurology | Tokyo Women’s Medical University | Department of Medicine, Kidney Center | Professor | Kosaku Nitta |
| Neurology | Nippon Medical School | Department of Nephrology | Professor | **Shuichi Tsuruoka** |

(3) Independent Efficacy and Safety Evaluation Committee

| Chairperson/Neurology | Kawasaki Medical School | Department of Nephrology and Hypertension | Professor | Naoki Kashihara |
| --- | --- | --- | --- | --- |
| Cardiovascular | National Cerebral and Cardiovascular Center | Department of Clinical Medicine and Development | Director | Masaharu Ishihara |
| Diabetes | Tokyo Medical University | Department of Diabetes, Endocrinology, Metabolism and Rheumatology | Professor | Masato Odawara |
| Diabetes | University of the Ryukyus | Second Department of Medicine, Division of Endocrinology, Diabetes and Metabolism, Hematology, Rheumatology | Professor | Hiroaki Masuzaki |
| Stroke | **Tokyo Women’s Medical University** | Department of Neurology | **Professor** | Kazuo Kitagawa |
| Gastroenterology | Osaka University | Department of Gastroenterology | **Associate Professor** | Masahiko Tsujii |

(4) Independent Data Monitoring Board

| Chairperson | Tokyo Medical University | Department of Cardiology | Professor | Akira Yamashina |
| --- | --- | --- | --- | --- |
|  | Jikei University School of Medicine | Division of Molecular Epidemiology | **Professor** | Mitsuyoshi Urashima |
|  | Dokkyo Medical University | Department of Cardiovascular Medicine, Nikko Medical Center | Professor | Takanori Yasu |

(5) Working Group

| FMD (representative) | Hiroshima University | Research Institute for Radiation Biology and Medicine | Professor | Yukihito Higashi |
| --- | --- | --- | --- | --- |
| FMD | Shimane University | Department of General Medicine | Professor | Yutaka Ishibashi |
| FMD | Osaka City University | **Department of Internal Medicine and Cardiology** | **Associate Professor** | Kenei Shimada |
| PWV/CAVI/AI | Tokyo Medical University | Department of Cardiology | Professor | Hirofumi Tomiyama |
| IMT | University of Tsukuba | **Department of Clinical Laboratory Medicine, Faculty of Medicine** | Instructor | Tomoko Ishizu |
| Echocardiography | Tokushima University | Department of Cardiovascular Medicine/Ultrasound Examination Center | Instructor | Hirotsugu Yamada |

(6) Measurement laboratories

Centralized measurement laboratories for IMT

**Department of Clinical Laboratory Medicine**, Faculty of Medicine, University of Tsukuba

Rationale for Amendment

Institutions and Titles were updated due to relocation and promotion.

***Ver. 6.0 documented on August 19, 2014***

*1. Dr. Shinichiro Ueda was assigned as a person in charge of the audit and removed from the management committee.*

Previous Text

23. Study Organization

(2) Steering committee

| Cardiovascular | Hokkaido University | Department of Cardiovascular Medicine | Professor | Hiroyuki Tsutsui |
| --- | --- | --- | --- | --- |
| Cardiovascular | Akita University | Department of Cardiovascular and Respiratory Medicine | Professor | Hiroshi Ito |
| Cardiovascular | Dokkyo Medical University | Department of Cardiovascular Medicine | Professor | Teruo Inoue |
| Cardiovascular | Showa University | Department of Diabetes, Metabolism, and Endocrinology | Professor | Tsutomu Hirano |
| Cardiovascular | Juntendo University | Department of Cardiovascular Medicine | Professor | Hiroyuki Daida |
| Cardiovascular | Mie University | Department of Cardiology and Nephrology | Professor | Masaaki Ito |
| Cardiovascular | National Cerebral and Cardiovascular Center | Department of Clinical Medicine and Development | Director | Masafumi Kitakaze |
| Cardiovascular | Tokushima University | Department of Cardiovascular Medicine | Professor | Masataka Sata |
| Cardiovascular | Nagasaki University | Department of Cardiovascular Medicine | Professor | Koji Maemura |
| **Hypertension** | **University of the Ryukyus Hospital** | **Clinical Research Support Center** | **Professor** | **Shinichiro Ueda** |
| Diabetes | Tokushima University | Diabetes Therapeutics and Research Center | Professor | Munehide Matsuhisa |
| Diabetes | Kawasaki Medical School | Department of Internal Medicine | Professor | Kohei Kaku |
| Metabolism | Yokohama City University | Department of Endocrinology and Metabolism | Professor | Yasuo Terauchi |
| Neurology | Tokyo Women’s Medical University | Department of Medicine, Kidney Center | Professor | Kosaku Nitta |
| Neurology | Nippon Medical School | Department of Nephrology | Professor | Shuichi Tsuruoka |

21. Audits

Audits are intended to determine whether or not this study is properly conducted and ensure the reliability of obtained data. The study organization will contract out audits on the study to an external party. When performing audits, records revealing the identities of the patients and secretes of medical information should be kept to protect privacy. The investigators and study sites should provide records on the study for direct access upon request and collaborate in this at the time of audits and investigations by the ethics review committee.

Revised Text

23. Study Organization

(2) Steering committee

| Cardiovascular | Hokkaido University | Department of Cardiovascular Medicine | Professor | Hiroyuki Tsutsui |
| --- | --- | --- | --- | --- |
| Cardiovascular | Akita University | Department of Cardiovascular and Respiratory Medicine | Professor | Hiroshi Ito |
| Cardiovascular | Dokkyo Medical University | Department of Cardiovascular Medicine | Professor | Teruo Inoue |
| Cardiovascular | Showa University | Department of Diabetes, Metabolism, and Endocrinology | Professor | Tsutomu Hirano |
| Cardiovascular | Juntendo University | Department of Cardiovascular Medicine | Professor | Hiroyuki Daida |
| Cardiovascular | Mie University | Department of Cardiology and Nephrology | Professor | Masaaki Ito |
| Cardiovascular | National Cerebral and Cardiovascular Center | Department of Clinical Medicine and Development | Director | Masafumi Kitakaze |
| Cardiovascular | Tokushima University | Department of Cardiovascular Medicine | Professor | Masataka Sata |
| Cardiovascular | Nagasaki University | Department of Cardiovascular Medicine | Professor | Koji Maemura |
| Diabetes | Tokushima University | Diabetes Therapeutics and Research Center | Professor | Munehide Matsuhisa |
| Diabetes | Kawasaki Medical School | Department of Internal Medicine | Professor | Kohei Kaku |
| Metabolism | Yokohama City University | Department of Endocrinology and Metabolism | Professor | Yasuo Terauchi |
| Neurology | Tokyo Women’s Medical University | Department of Medicine, Kidney Center | Professor | Kosaku Nitta |
| Neurology | Nippon Medical School | Department of Nephrology | Professor | Shuichi Tsuruoka |

21. Audits

Audits are intended to determine whether or not this study is properly conducted and ensure the reliability of the data. The study organization will contract out audits to an external party. When performing audits, records revealing the identities of patients and confidential medical information should be kept to protect privacy. The investigators and study sites should provide records on the study for direct access upon request and collaborate in this at the time of audits and investigations by the ethics review committee.

**Audit manager: Shinichiro Ueda, Director, Clinical Research Support Center, University of the Ryukyus Hospital**

Rationale for Amendment

Audits is newly added and mentioned. Dr. Shinichiro Ueda became the audit manager and he was removed from members of steering committee.

*2. The institution of Dr Masaharu Ishihara changed.*

Previous Text

23. Study Organization

(3) Independent Efficacy and Safety Evaluation Committee

| Chairperson/Neurology | Kawasaki Medical School | Department of Nephrology and Hypertension | Professor | Naoki Kashihara |
| --- | --- | --- | --- | --- |
| **Cardiovascular** | **National Cerebral and Cardiovascular Center** | **Department of Clinical Medicine and Development** | **Director** | **Masaharu Ishihara** |
| Diabetes | Tokyo Medical University | Department of Diabetes, Endocrinology, Metabolism and Rheumatology | Professor | Masato Odawara |
| Diabetes | University of the Ryukyus | Second Department of Medicine, Division of Endocrinology, Diabetes and Metabolism, Hematology, Rheumatology | Professor | Hiroaki Masuzaki |
| Stroke | Tokyo Women’s Medical University | Department of Neurology | Professor | Kazuo Kitagawa |
| Gastroenterology | Osaka University | Department of Gastroenterology | Associate Professor | Masahiko Tsujii |

Revised Text

23. Study Organization

(3) Independent Efficacy and Safety Evaluation Committee

| Chairperson/Neurology | Kawasaki Medical School | Department of Nephrology and Hypertension | Professor | Naoki Kashihara |
| --- | --- | --- | --- | --- |
| **Cardiovascular** | **Hyogo College of Medicine** | **Division of Cardiovascular Medicine and Coronary Heart Disease** | **Professor** | **Masaharu Ishihara** |
| Diabetes | Tokyo Medical University | Department of Diabetes, Endocrinology, Metabolism and Rheumatology | Professor | Masato Odawara |
| Diabetes | University of the Ryukyus | Second Department of Medicine, Division of Endocrinology, Diabetes and Metabolism, Hematology, Rheumatology | Professor | Hiroaki Masuzaki |
| Stroke | Tokyo Women’s Medical University | Department of Neurology | Professor | Kazuo Kitagawa |
| Gastroenterology | Osaka University | Department of Gastroenterology | Associate Professor | Masahiko Tsujii |

Rationale for Amendment

The institution of Dr Masaharu Ishihara changed.

***Ver. 6.1 documented on January 27, 2015***

***(as final version in English)***

*1. The end of implementation period extended from “Sept. 30 2015” to “Sept. 30 2016”.*

Previous Text

13. Period of the Study

Date of approval of the ethics review committee to **September 30, 2015**

Treatment observation period: December 31, 2014 (closing date of enrollment: September 30, 2012)

Study period: **September 30, 2015** (publication of analysis and study results)

Revised Text

13. Period of the Study

Date of approval of the ethics review committee to **September 30, 2016**

Treatment observation period: December 31, 2014 (closing date of enrollment: September 30, 2012)

Study period: **September 30, 2016** (publication of analysis and study results)

Rationale for Amendment

The period of the study is extended to September 30, 2016.

**Summary of changes**

| Protocol　Version No. | Summary of Changes |
| --- | --- |
| 1  September 19, 2010 | Initial application |
| 2  April 11, 2011 | Amendment 1 leading to Protocol v2.0.  This amendment included:   - Exclusion criteria: “a patient using GLP-1 analogue”, “a patient with malignant tumor” added. - Combination therapy with sitagliptin and alpha-GI drug is under discussion for approval and will be available after the approval of Ministry of Health, Labour and Welfare in Japan. - “Use of HMG-CoA Reductase Inhibitor“ was added to random allocation factors - “Use of GLP-1 analogue” was prohibited in sitagliptin group. - Amylase was added to measure in “8. Observations and Tests (7) Blood biochemistry”. Other explanations were added. - Roche Diagnostics K.K. joined to measure NT-proBNP and Fujirebio Inc joined to measure high molecular weight adiponectin as Joint study sites. (23.Study Organization (8) Joint study sites). - Yasunori Sato joined as a member of Statistical Analysis Committee (23.Study Organization (7) Statistical Analysis Committee). - Hirotsugu Yamada joined as a member of working group of echocardiography (23.Study Organization (5) Working Group). |
| 3  May 1, 2012 | Amendment 2 leading to Protocol v3.0.  Protocol 2.0 was amended to allow:   - Combination therapy with sitagliptin and alpha-GI drug is now available after approval of Ministry of Health, Labour and Welfare in Japan. - The primary endpoint was defined from “amount of change and percentage change in common carotid artery” to “percentage change in common carotid artery”. “amount of change in common carotid artery” was defined in secondary endpoint. - Interim analysis is performed at one year after the registration of a final patient. |
| 4  June 19, 2012 | Amendment 3 leading to Protocol v4.0  Protocol 3.0 was amended to allow:   - “15. Target Sample Size and Rationale for Determination” was modified. - The end of implementation period extended from “June 30 2014” to “Sept 30 2014”. - Takanori Yasu joined as a member of “Independent Data Monitoring Board” (23.Study Organization (4) Independent Data Monitoring Board). |
| 4.1  February 22, 2013 | Amendment 3 leading to Protocol v4.1  Protocol 4.0 was amended to allow:   - One of Research secretariats (Dr. Kazuhisa Kodama) was deleted due to the relocation. |
| 5  April 1, 2014 | Amendment 4 leading to Protocol v5.0:  Protocol 4.0 was amended to allow   - The end of implementation period extended from “Sept. 30 2014” to “Sept. 30 2015”. - Audit was spitulated. |
| 6  August 19, 2014 | Amendment 5 leading to Protocol v6.0:  Protocol 5.0 was amended to allow:   - Dr. Shinichiro Ueda was assigned as a person in charge of the audit and removed from the management committee. |
| 6.1  January 27, 2015 | Amendment 6 leading to Protocol v6.1:  Protocol 6.0 was amended to allow:   - The end of implementation period extended from “Sept. 30 2015” to “Sept. 30 2016”. |
