# Supplementary material for: The Effect of Sitagliptin on Carotid Artery Atherosclerosis in Type 2 Diabetes: The PROLOGUE Randomized Controlled Trial
Source: PLoS Med. 2016 Jun 28;13(6):e1002051. doi: 10.1371/journal.pmed.1002051 (PMC4924847; doi:10.1371/journal.pmed.1002051)
Supplement: S4 Text — (DOCX) [file pmed.1002051.s008.docx]

**Organization of PROLOGUE study**

**1. Chief Investigator**

Koichi Node (Department of Cardiovascular Medicine, Saga University).

**2. Principal Investigators**

Koichi Node (Department of Cardiovascular Medicine, Saga University); Toyoaki Murohara (Department of Cardiology, Nagoya University Graduate School of Medicine)

**3. Steering Committee**

Koichi Node (Department of Cardiovascular Medicine, Saga University); Toyoaki Murohara (Department of Cardiology, Nagoya University Graduate School of Medicine); Hiroyuki Tsutsui (Department of Cardiovascular Medicine, Hokkaido University Graduate School of Medicine); Hiroshi Ito (Department of Cardiovascular and Respiratory Medicine, Akita University Graduate School of Medicine); Teruo Inoue (Department of Cardiovascular Medicine, Dokkyo Medical University); Tsutomu Hirano (Department of Diabetes, Metabolism, and Endocrinology, Showa University School of Medicine); Hiroyuki Daida (Department of Cardiovascular Medicine, Juntendo University Graduate School of Medicine); Masaaki Ito (Department of Cardiology and Nephrology, Mie University Graduate School of Medicine); Masafumi Kitakaze (Department of Clinical Medicine and Development, National Cerebral and Cardiovascular Center); Masataka Sata (Department of Cardiovascular Medicine, Institute of Biomedical Sciences, Tokushima University Graduate School); Koji Maemura (Department of Cardiovascular Medicine, Nagasaki University Graduate School of Biomedical Sciences); Munehide Matsuhisa (Diabetes Therapeutics and Research Center, Tokushima University); Kohei Kaku (Department of Internal Medicine, Kawasaki Medical School); Yasuo Terauchi (Department of Endocrinology and Metabolism, Yokohama City University); Kosaku Nitta (Department of Medicine, Kidney Center, Tokyo Women's Medical University); Shuichi Tsuruoka (Department of Nephrology, Nippon Medical School); Hirofumi Tomiyama (Department of Cardiology, Tokyo Medical University); Tomoko Ishizu (Department of Clinical Laboratory Medicine, Faculty of Medicine, University of Tsukuba); Yukihito Higashi (Department of Cardiovascular Regeneration and Medicine, Research Institute for Radiation Biology and Medicine, Hiroshima University); Yutaka Ishibashi (Department of General Medicine, Shimane University Faculty of Medicine); Kenei Shimada (Department of Internal Medicine and Cardiology, Osaka City University Graduate School of Medicine); Hirotsugu Yamada (Department of Cardiovascular Medicine, Tokushima University Hospital).

**4. Independent Efficacy and Safety Evaluation Committee**

Naoki Kashihara (Department of Nephrology and Hypertension, Kawasaki Medical School); Masaharu Ishihara (Division of Cardiovascular Medicine and Coronary Heart Disease, Hyogo College of Medicine); Masato Odawara (Department of Diabetes, Endocrinology, Metabolism and Rheumatology, Tokyo Medical University); Hiroaki Masuzaki (Second Department of Medicine, Division of Endocrinology, Diabetes and Metabolism, Hematology, Rheumatology, Graduate School of Medicine, University of the Ryukyus); Kazuo Kitagawa (Department of Neurology, Tokyo Women's Medical University); Masahiko Tsujii (Department of Gastroenterology, Osaka Rosai Hospital).

**5. Independent Data Monitoring Committee**

Akira Yamashina (Department of Cardiology, Tokyo Medical University); Takanori Yasu (Department of Cardiovascular Medicine, Dokkyo Medical University Nikko Medical Center); Mitsuyoshi Urashima (Division of Molecular Epidemiology, Jikei University School of Medicine).

**6. Independent Audit Team**

Shinichiro Ueda (Department of Clinical pharmacology & Therapeutics, University of the Ryukyus); Yumi Ikehara (Clinical Research and Quality Management Center, University of the Ryukyus); Satomi Tanabe (Clinical Research Supporting Center, University of the Ryukyus Hospital).

**7. Participating Institutions and Hospitals**

Saga University; Akita University Graduate School of Medicine; Tokyo Medical University; Juntendo University, Graduate School of Medicine; Tokushima University; Osaka University Graduate School of Medicine; Toyota Memorial Hospital; University of Tsukuba; Showa University School of Medicine; National Cerebral and Cardiovascular Center; Mie University Graduate School of Medicine; Hiroshima City Hospital; Nagoya University Graduate School of Medicine; Kawasaki Medical School; Tsushima Municipal Hospital; Nagoya Ekisaikai Hospital; Nagasaki University Graduate School of Biomedical Sciences; Omura Municipal Hospital; Komaki City Hospital; Tokyo Women's Medical University; Dokkyo Medical University; Kasugai Municipal Hospital; Japanese Red Cross Nagoya Daiichi Hospital; Nishio Municipal Hospital; Mitsubishi Nagoya Hospital; Nagoya central hospital; Nagoya Daini Red Cross Hospital; Fujita Health University Banbuntane Hotokukai Hospital; Anjo Kosei Hospital; Hokusho Central Hospital; Gifu Prefectural Tajimi Hospital; Hiroshima University; Tosei General Hospital; Nagoya Kyoritsu Hospital; Sasebo Chuo Hospital; Nakatsugawa Municipal Hospital; National Hospital Organization Nagoya Medical Center; Tokyo Medical University Ibaraki Medical Center; Handa City Hospital; Miyazaki Medical Association Hospital; Eguchi Hospital; Saga-ken Medical Centre Koseikan; Meijo Hospital; Hiramatsu Hospital; Yamamoto Memorial Hospital; Kanto Rosai Hospital; Saga City Fuji-Yamato Spa. Hospital; Karatsu Red Cross Hospital
