# Supplementary material for: The Effect of Sitagliptin on Carotid Artery Atherosclerosis in Type 2 Diabetes: The PROLOGUE Randomized Controlled Trial
Source: PLoS Med. 2016 Jun 28;13(6):e1002051. doi: 10.1371/journal.pmed.1002051 (PMC4924847; doi:10.1371/journal.pmed.1002051)
Supplement: S6 Text — (DOCX) [file pmed.1002051.s010.docx]

**Competing Interests**

MA declares no competing interests. YKB received honoraria from AstraZeneca, Mitsubishi Tanabe, MSD, and Takeda. YKB received research grant from AstraZeneca, Daiichi Sankyo, Mitsubishi Tanabe, MSD, and Takeda. KD declares no competing interests. YH declares no competing interests. TIn declares no competing interests. MI received honoraria from Abbott Vascular Japan, Amgen, Astellas, AstraZeneca, Bayer, Boehringer Ingelheim, Bristol-Myers, Chugai, Daiichi Sankyo, Eisai, Kissei, Kyowa Hakko Kirin, Mochida, MSD, Novartis, Ono, Otsuka, Sanofi, Sanwa Kagaku Kenkyusho, and Toa Eiyo. MI received research funding from Abbott Vascular Japan, Astellas, Bayer, Boehringer Ingelheim, Boston Scientific Japan, Daiichi Sankyo, Eisai, Fukuda Denshi, Goodman, MID, Mitsubishi Tanabe, Mochida, MSD, Novartis, Ono, Pfizer, Sanofi, and Teijin Pharma. TIs declares no competing interests. KKa received honoraria from Astellas, AstraZeneca, Bayer, Boehringer Ingelheim, Fuji-Film Pharma, Kissei, Kowa, Mitsubishi Tanabe, MSD, Novartis, Novo Nordisk, Sanofi, Sanwa Kagaku Kenkyusho, Sumitomo Dainippon, Taisho Toyama, and Takeda. KKa received research grants from Astellas, Boehringer Ingelheim, Daiichi Sankyo, Taisho Pharmaceutical, and Takeda. HK declares no competing interests. NK received honoraria from Astellas, Daiichi Sankyo, MSD, Otsuka, Shionogi, Sumitomo Dainippon, and Takeda. NK received research grant from Astellas, Bayer, Daiichi Sankyo, MSD, Otsuka, Sanwa Kagaku Kenkyusho, Shionogi, Sumitomo Dainippon, and Takeda. KKi received honoraria from AstraZeneca, Bayer, Boehringer Ingelheim, Bristol-Myers, Daiichi Sankyo, Otsuka, Pfizer, Sanofi, and Shionogi. KKi received research grants from Boehringer Ingelheim, Daiichi Sankyo, Otsuka, and Sanofi. MK received honoraria from Abbott Vascular Japan, Asahi Kasei Medical, Astellas, AstraZeneca, Bayer, Boehringer Ingelheim, Daiichi Sankyo, Fujifilm, Japan Medical Data, Kowa, Kyowa Hakko Kirin, Mitsubishi Tanabe, MSD, Novartis, Novo Nordisk, Ono, Otsuka, Pfizer, Sanofi, Sawai, Shionogi, Sumitomo Dainippon, and Takeda. MK received research grants from Abbott Vascular Japan, Calpis, Japan Cardiovascular Research Foundation, Japanese government, Japan Heart Foundation, Mitsubishi Tanabe, Nihon Kohden, Novartis, Ono, Otsuka, Pfizer, Sanofi, and Takeda. KM received honoraria from Boehringer Ingelheim, Kowa, Kyowa Hakko Kirin, Mitsubishi Tanabe, MSD, Novartis, Ono, Sanwa Kagaku Kenkyusho, and Takeda. KM received research grants from Boehringer Ingelheim, Kowa, Mitsubishi Tanabe, MSD, Novartis, Sanwa Kagaku Kenkyusho, and Takeda. MM received honoraria from Astellas, Eli Lilly, Mitsubishi Tanabe, Novo Nordisk, Sanofi, and Takeda. MM received research grant from Astellas, Nihon Unisys, Nikkiso, and Terumo. TM received honoraria from Bayer, Boehringer Ingelheim, Daiichi Sankyo, Kowa, Mitsubishi Tanabe, MSD, Pfizer, Sumitomo Dainippon, and Takeda. TM received research grant from Astellas, Boehringer Ingelheim, Daiichi Sankyo, Kowa, Mitsubishi Tanabe, MSD, Novartis, Otsuka, Pfizer, Sanofi, Sumitomo Dainippon, Takeda, and Teijin Pharma. MN declares no competing interests. KN received honoraria from Astellas, Boehringer Ingelheim, Daiichi Sankyo, Merck, Mitsubishi Tanabe, Sanofi, and Takeda. KN received research funding from Astellas, Boehringer Ingelheim, Grant-in-Aid for Scientific Research from the Ministry of Education, Culture, Sports, Science and Technology in Japan, Mitsubishi Tanabe, Sanwa Kagaku Kenkyusho, Takeda, and Teijin Pharma. JO belongs to the research program faculty (chair course) sponsored by Fukuda Denshi. MS received honoraria from MSD. MS received research grant from MSD and Ono. YS received honoraria from Elekta, Pfizer, and Siemens. YS received research grant from Kowa. AT declares no competing interests. HT received honorarium from Omron Health Care. SU received honoraria from Boehringer Ingelheim, Daiichi Sankyo, MSD, and Pfizer. SU received research grant from Bayer, Bristol Myers Squibb, Kowa, MSD, Pfizer, and Takeda. HY received honoraria from MSD. HY received research grant from MSD and Ono. KY declares no competing interests.
